# The many dimeric faces of Lys49 PLA_2_‐like proteins: Conformational plasticity and membrane binding drive functional dimer states

**DOI:** 10.1002/pro.70449

**Published:** 2026-01-20

**Authors:** Diane C. A. Lima, Vinicius Firmino dos Santos, Bernardo Rassi, Richard J. Ward, Thereza A. Soares

**Affiliations:** ^1^ Department of Chemistry, FFCLRP University of São Paulo SP Brazil; ^2^ Hylleraas Centre for Quantum Molecular Sciences University of Oslo Oslo Norway

**Keywords:** conformational heterogeneity and membrane binding, FRET efficiency, multiscale molecular dynamics simulations, phospholipases A2, tryptophan fluorescence anisotropy

## Abstract

Lys49 secreted phospholipase A_2_‐like proteins (sPLA_2_s) are major myotoxins in viperid snake venoms, causing rapid muscle damage in envenomation. Beyond their clinical relevance, these small non‐catalytic proteins provide a model to study how quaternary structure and conformational dynamics enable catalysis‐independent membrane disruption. Using site‐directed mutagenesis, fluorescence anisotropy, and extensive atomistic and coarse‐grained molecular dynamics simulations, we characterized the conformational landscape of Bothropstoxin‐I (BthTx‐I), a prototypical Lys49 sPLA_2_‐like protein. Our results show that compact and extended dimers coexist in solution but differ in flexibility, with only the extended dimer reproducing experimental FRET efficiencies across wild‐type and mutant proteins. Atomistic MD simulations reveal that the extended dimer undergoes hinge‐like motions that preserve quaternary structure while sampling substates compatible with membrane engagement. Coarse‐grained simulations demonstrate that only geometries similar to the extended crystallographic conformation allow both C‐terminal loops to simultaneously insert into the bilayer, stabilizing the membrane‐bound state required for phospholipid disruption. These findings resolve the long‐standing debate over compact versus extended dimer assemblies by demonstrating that the extended conformation is the functionally competent state, providing a unifying mechanistic framework that links quaternary structure dynamics to the molecular basis of myotoxicity. By pinpointing the structural features essential for productive membrane engagement, this work establishes a predictive platform that is expected to accelerate the rational design of next‐generation inhibitors for more effective treatment of snakebite envenomation.

## 1 INTRODUCTION

Phospholipases A2 (PLA_2_‐EC 3.1.1.4) act at the water/membrane interface and catalyze the hydrolysis of the sn‐2 acyl bonds of sn‐3 glycerophospholipids (Mouchlis and Dennis 2022; Van Deenenl and de Haas 1963). The physico‐chemical and biological effects of PLA_2_s on membranes are typically associated with alteration in the composition due to the depletion of phospholipids and accumulation of hydrolysis products (Khan and Ilies 2023). Lysophospholipids generated by PLA_2_ contribute to modulation of membrane organization and cell signaling, and the fatty acid products may themselves be bioactive, or may serve as precursors for the synthesis of other bioactive compounds such as prostaglandin and leukotriene inflammatory mediators (Murakami 2025).

Based on structural features, the PLA_2_ superfamily comprises 15 groups (Dennis et al. 2011; Schaloske and Dennis 2006), and the secreted group I/II (GI/II) PLA_2_s are low‐molecular‐weight Ca^2+^‐dependent enzymes with a His/Asp catalytic dyad that are found in a wide variety of biological fluids and cells (Murakami et al. 2015), including snake venoms where they display a wide range of pharmacological effects such as inflammation, necrosis, myonecrosis, hemolysis, and anticoagulation (Sampat et al. 2023). A general consensus mechanism suggests that the venom sPLA_2_ interacts with sites on the target cell membrane, evoking cellular responses that result in the toxic effects (Neess et al. 2015). Thus, catalytic and non‐catalytic events may both be relevant for understanding a given toxin function (Lomonte and Rangel 2012; Saikia et al. 2011).

The Lys49 sPLA_2_‐like proteins are non‐catalytic Group II venom sPLA_2_s present in viperid snake venoms which demonstrate potent myotoxic effects (Lomonte 2023; Ward et al. 2002). Loss of the catalytic function results from substitution of the Asp49 residue of the catalytic Asp/His dyad by a Lys, where the ε‐amino group occupies the volume normally filled by the Ca^2+^ ion cofactor (Arni et al. 1995; Holland et al. 1990). Although lacking catalytic activity, the Lys49 sPLA_2_‐like proteins show a membrane permeabilizing activity against model liposome membranes (de Oliveira et al. 2001a; Díaz et al. 1991; Rufini et al. 1992) and cultured myocyte membranes (Villalobos et al. 2007). The Lys49 sPLA_2_‐like proteins target the muscle cell plasma membrane, where membrane permeabilization leads to a large and unregulated Ca^2+^ influx and myotoxicity (Cintra‐Francischinelli et al. 2009; Gutiérrez and Ownby 2003). Understanding the basis of the catalysis independent membrane permeabilization is therefore important for understanding the myotoxic activity of the Lys49 sPLA_2_‐like proteins, and this knowledge can be applied to the development of anti‐ophidic drugs for the treatment of snake bites (Salvador et al. 2024).

**FIGURE 1:**
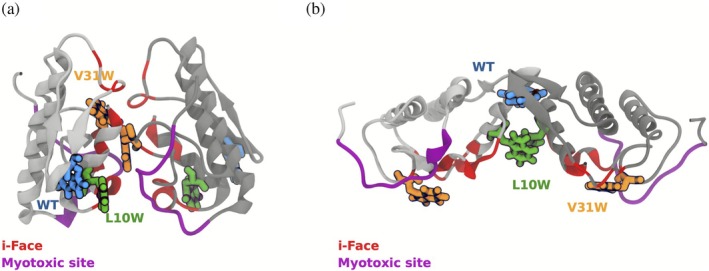
Cartoon representation of two possible dimeric conformations of bothropstoxin I (BthTxI) obtained from the asymmetric unit in the 3HZD entry of the RCSB PDB database. The positions of the Trp residues at position 77 in the wild‐type, W77H/L10W and W77H/V31W variants are shown for the (a) compact and (b) extended conformations of the homodimer. In the compact conformation the Trp31 of both monomers are located at the homodimer interface, whereas in the extended conformation the Trp77 (the position in the wild‐type) and W10 are located at the homodimer interface. The location of the C‐terminal loops of the monomers is shown in fuchsia.

In common with other secreted GI/II PLA_2_, the Lys49 sPLA_2_‐like proteins present a hydrophobic substrate binding cleft leading to the active site (Figure 1) (Ward et al. 1998), and this region together with the surrounding residues defines the surface of the protein that associates with the membrane, denominated as the interfacial binding site (Pieterson et al. 1974) or i‐face (Ramirez and Jain 1991; Winget et al. 2006). The key role of the i‐face in snake venom sPLA_2_s interactions with target membranes has focused drug binding studies to this region (Xiao et al. 2017). In Lys49 sPLA_2_‐like proteins, the i‐face is rich in cationic Arg and Lys residues and is mirrored by the requirement of anionic lipids in liposome model membranes for the manifestation of bilayer permeabilization (de Oliveira et al. 2001a; Díaz et al. 1991; Ferreira and Ward 2009; Rufini et al. 1992; Ward et al. 2002). In addition, the cationic C‐terminal loop region of the Lys49 sPLA_2_‐like proteins has been demonstrated to be involved in both membrane permeabilization and in the myotoxic activity (Lomonte 2023; Lomonte et al. 2003). The C‐terminal loop is a key feature defining the so‐called “myotoxic site” (Figure 1) (Lomonte et al. 1994), although it should be noted that alanine scanning mutagenesis has identified regions outside the C‐terminal loop as being important for myotoxicity, suggesting a structural overlap between the interface binding and myotoxic sites (Aragão et al. 2009). The strong correlation of the shared structural features between the model membrane permeabilization and myotoxic effects is in accord with the consensus proposal for myotoxic activity and has led to the widespread use of liposome model membranes to study the Ca^2+^‐independent membrane damaging activity of the Lys49 sPLA_2_‐like proteins.

Amphiphile binding to the i‐face has been suggested to induce structural changes that underlie the significant increase in catalytic activity observed on PLA_2_s association with membranes (Castro‐Amorim et al. 2023; Tatulian 2001). In the Lys49 sPLA_2_‐like proteins, structural changes on ligand binding to the i‐face and hydrophobic channel can result in conformation alterations in the C‐terminal loop (Ambrosio et al. 2005; Ullah et al. 2012), which have been suggested to result in allosteric activation of myotoxic activity (dos Santos et al. 2009; Fernandes et al. 2013). These studies highlight the importance of the i‐face; however, details of the Lys49 sPLA_2_‐like protein/membrane interaction are controversial, and key events such as the details of the interactions of i‐face residues with membrane phospholipids and the consequences of Lys49 sPLA_2_‐like protein binding on the organization of the membrane bilayer remain obscure.

The bothropstoxin‐I (BthTx‐I) is a Lys49 sPLA_2_‐like protein present in the venom of the viperid snake *Bothrops jararacuçu* (Homsi‐Brandeburgo et al. 1988). Two models have been proposed for the Lys49 sPLA_2_‐like protein/membrane interaction (Figure 1). The “hinge” model is based on the observation of two conformational states of an extended BthTx‐I homodimer using x‐ray crystallography (Figure 1b) (da Silva Giotto et al. 1998). In this model, a homodimer interface is stabilized by reciprocal intermolecular hydrogen bonding between a highly conserved Glu12/Trp77/Lys80 triad that brings the β‐wing regions of each monomer into contact. It is suggested that conformational flexibility at this dimer interface acts as a hinge that permits variation in the angle between the two monomers and that these structural transitions in the membrane‐bound homodimer result in the insertion of the C‐terminal loop region into the membrane bilayer.

As the x‐ray structures of more Lys49 sPLA_2_‐like proteins complexed with ligands have become available, an alternative interpretation of the crystallographic data based on a more compact homodimer has been suggested, in which the protein–protein interface is defined by contacts between the calcium‐binding and C‐terminal loops (Figure 1a). The suggested mechanism of this compact homodimer involves a proposed “two angle model” (Fernandes et al. 2013), in which fatty acid binding to the substrate binding cleft induces an alteration in the relative orientation between the two monomers and creates an extended cationic patch on the dimer surface that favors membrane docking. This interaction results in membrane destabilization by penetration of hydrophobic residues in the C‐terminal loop. The fatty acid binding event would then induce an allosteric alteration in the conformation of the C‐terminal loop that exposes a hydrophobic patch which is proposed to further increase the membrane affinity of this region (Fernandes et al. 2013) and explain the synergistic myotoxic effects observed between Asp49 and Lys49 sPLA_2_‐like proteins (Cintra‐Francischinelli et al. 2009; Mora‐Obando et al. 2014). Results from small angle x‐ray scattering (SAXS) experiments have been cited in support for the compact dimer conformation, which forms the basis of the “two angle model” model (Murakami et al. 2007). However, the SAXS experiments did not address conformational heterogeneity or sample polydispersity, leaving open the possibility that the reconstructed molecular envelopes represent averaged features of multiple coexisting quaternary states rather than a single, biologically relevant conformation.

Certain shared features of the “hinge” and “two angle model” are apparent. Both models are based on alternative interpretations of homodimeric structures observed in the majority of the x‐ray crystal structures of Lys49‐PLA_2_s. Furthermore, both models present a viable explanation for contact of the C‐terminal loop region with the target membrane. It is also noteworthy that the monomers in either dimer configuration can bind fatty acids and undergo allosteric activation. Nevertheless, although both models propose quaternary structural changes in the membrane bound form, the protein surfaces involved in the protein/membrane interaction and nature of the structural transitions differ significantly.

Despite these efforts, the structural basis of Lys49 sPLA_2_‐like protein myotoxicity remains unresolved, particularly regarding whether a single quaternary arrangement or multiple conformations underlie the functional mechanism. The reliance on x‐ray crystallographic snapshots and ambiguities in the interpretation of low‐resolution SAXS envelopes has left fundamental questions unanswered about the extent of conformational heterogeneity in solution and its role in membrane binding. In this work, we combine atomistic and coarse‐grained molecular dynamics simulations with analysis of experimental anisotropy data to dissect the conformational landscapes of alternative dimeric assemblies. Our results reveal that distinct quaternary states not only coexist in solution but also differ in their ability to sustain productive membrane interactions, providing a unifying framework that reconciles crystallographic variability with functional myotoxic activity.

## 2 RESULTS AND DISCUSSION

### 2.1 Combined experimental intrinsic tryptophan fluorescence emission anisotropy and MD‐derived energy transfer analyses support an extended dimer conformation in solution

**FIGURE 2:**
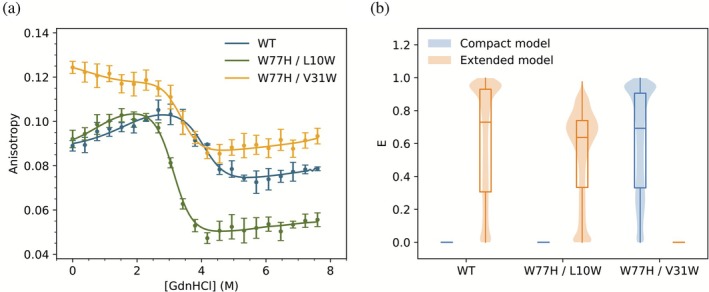
(a) Tryptophan fluorescence emission anisotropy with increasing guanidinium hydrochloride (GdnHCl) concentration for the wild‐type BthTx‐I and the double mutants W77H/L10W and W77H/V31W (colors as shown in the figure panel). The solid curves are derived from least squares fitting the anisotropy data using a three‐state denaturation model of a BthTx‐I homodimer, and the best fit parameters are presented in Table 1. Further details of the tryptophan fluorescence emission anisotropy measurements and curve fitting are provided in section 3. (b) Calculated energy transfer efficiency (*E*) between tryptophan side chains calculated for MD simulations of the compact and extended dimer models, respectively. The analysis is described in section 3, and the scripts used are provided in the Data Availability section. Statistics were calculated for three independent simulations of each protein. A total of 9 μs production MD simulations were used.

The denaturation of wild‐type BthTx‐I and the single tryptophan W77H/L10W and W77H/V31W variants by guanidinium hydrochloride (GdnHCl) was monitored by tryptophan fluorescence emission anisotropy (Figure 2a). The denaturation curves of all proteins showed three phases: either a gradual increase or decrease in the fluorescence anisotropy over the (GdnHCl) of 0–2.5M, a more rapid decrease in the fluorescence anisotropy was observed between 2.5 and 4M (GdnHCl), followed by a gradual increase in fluorescence anisotropy with increasing (GdnHCl) concentration in the range 4–7.6M. These three phases in the fluorescence anisotropy curves result from conformation transitions during protein unfolding, and the solid curves in Figure 2a were fitted using a three‐state denaturation model as previously applied to BthTx‐I (Ruller et al. 2003). In this model, the first fluorescence anisotropy transition at low (GdnHCl) is the consequence of the dissociation of the BthTx‐I homodimer to yield two folded BthTx‐I monomers, which denature at higher (GdnHCl) to yield two unfolded BthTx‐I monomers. The model predicts that the dimer to monomer equilibrium is dependent on the protein concentration, and the native monomer to denatured monomer equilibrium is independent of protein concentration. These predictions, together with identification of the intermediate protein conformations, have been experimentally confirmed in the case of chemical denaturation of BthTx‐I (Ruller et al. 2003).

It is noteworthy that the three‐state model is valid for both the compact and extended homodimers; however, further analysis of the fluorescence anisotropy curves can provide key information to discriminate between the two alternative dimer conformations. The measured intrinsic tryptophan fluorescence anisotropy of the BthTxI will be influenced not only by the rotation of the individual indole side chains but also by the rotation of the protein, where smaller proteins that rotate faster show reduced fluorescence anisotropy as compared to larger proteins. These molecular motions will depolarize the fluorescence emission and so reduce the intrinsic tryptophan fluorescence anisotropy. On the basis of molecular rotation alone, it would be expected that the dimer to folded monomer conformation transition would reduce the measured fluorescence anisotropy. The observed initial reduction in the intrinsic tryptophan fluorescence anisotropy in the W77H/V31W variant (Figure 2a) is readily explained by increased monomer rotation as a result of dimer dissociation, and the subsequent anisotropy decrease at higher (GdnHCl) is attributed to increased indole side chain rotation as the monomer unfolds. In contrast, the dimer to folded monomer transition in the wild‐type BthTxI and the W77H/L10W variant is associated with an increase in the intrinsic tryptophan fluorescence anisotropy, showing that molecular rotation alone cannot explain the experimental observation and that an additional depolarizing effect is present in the protein dimers which is absent in the monomers. This effect has been previously observed in the BthTxI and has been attributed to Förster resonance energy transfer (FRET) between the tryptophan residues that are in close proximity at the dimer interface assuming the extended dimer configuration (de Oliveira et al. 2001b; Ruller et al. 2003; Ruller et al. 2005). The molecular dynamics (MD) simulations in the present study generate the atomic coordinates of the indole side chains of all variants in both the compact and extended configurations and permit a critical evaluation of the fluorescence anisotropy results.

The Förster resonance energy transfer efficiency (*E*) between the tryptophan side chains in all single tryptophan variants in both the compact and extended dimer configurations was calculated from the indole side chain atomic coordinates in the MD simulations (Figure 2b). The efficiency, *E*, provides a metric of spatial proximity, orientation, and structural dynamics of the tryptophan residues in the variants. Efficient energy transfer (*E* > 0.5) only occurs when the tryptophan residues are in close spatial proximity (typically within ~10 Å; Figure S3, Supporting Information) and adopt optimal relative orientations. Atomistic MD simulations, totaling 9 μs of production time across three independent replicas for BthTx‐I and variants, revealed drastic differences in energy transfer efficiency (*E*) between the compact and extended conformations (Figure 2b). The extended conformation of the wild‐type and W77H/L10W variant has high calculated transfer efficiencies, whereas the compact structures have near zero efficiencies (Figure 2b). In contrast, the W77H/V31W variant displays the inverse trend, showing near zero calculated transfer efficiency in the extended and high transfer efficiency in the compact configuration (Figure 2b). The high transfer efficiency predicted in the extended conformation of the wild‐type and W77H/L10W variant is the consequence of the close proximity between tryptophans at the dimer interface in the extended dimer conformation (Figure 1) and is consistent with the reduction in the experimentally observed fluorescence emission anisotropy observed in the dimeric form of these proteins (Figure 2a).

### 2.2 Conformational heterogeneity of the extended BthTx‐I conformation

**FIGURE 3:**
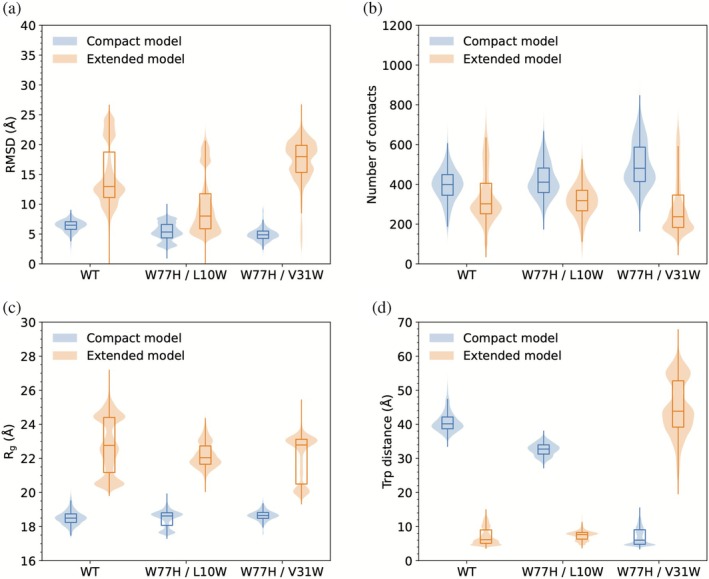
(a) Average atom‐positional RMSD of the backbone atoms (C, Ca, Cb, N) for all residues with respect to the two dimer arrangement in the X‐ray structure (PDB ID 3HZD). (b) Inter‐monomer average number of atomic contacts (*r* < 4.5 Å). (c) Average radius of gyration (*R*_*g*_) calculated for all atoms. (d) Average pair distance between center of geometry of Trp side chains for simulations of the Compact and Extended models, respectively. Statistics were calculated for three independent simulations of each protein. Time‐series for these properties are available in Supporting Information.

We compared the structural dynamics of both the compact and extended homodimer conformations in atomistic MD simulations (Figure 3). The root‐mean‐square deviation (RMSD) was calculated for the entire dimer after first superposing the backbone atoms of one monomer onto its crystallographic coordinates (Figures 3a and S4). Since the BthTx‐I monomer contains seven intramolecular disulfide bonds, large segments of the polypeptide main‐chain are constrained in a nearly invariant fold and this superposition procedure ensures that the calculated RMSD is dominated by the relative motions between the monomers in the dimeric conformations. It is important to note that the RMSD does not directly measure thermodynamic stability, but rather reports by how much the sampled ensemble deviates from the reference x‐ray crystal‐lattice structure. Crystal packing forces, low temperature, and dehydration effects in protein x‐ray crystallography experiments can favor conformations that are not fully representative of the ensemble in aqueous solution under physiological conditions. For this reason, the calculated RMSD primarily reflects solution‐phase inter‐domain motions within the dimer rather than loss of the native protein fold.

The compact and extended dimer conformations exhibit distinct behaviors in the atomistic MD simulations. The compact dimers of the wild‐type BthTx‐I and variants exhibit low average RMSD values and constant *R*_*g*_ values of 18–19 Å (Figure 3a), indicating only minor inter‐monomer conformational changes between the simulations. The number of contacts between the two monomers is also maintained indicating a stable and well‐packed dimer interface. In contrast, the variable RMSD values of the extended dimer conformation indicate a greater inter‐monomer rearrangement with respect to the crystal structure (Figures 3a and S4). The conserved average inter‐monomer contact number and average distance between the Trp pairs during the simulations suggest that this conformational variability occurs with the retention of the extended dimer quaternary structure (Figures 3b,d, S5, and S7). This interpretation is supported by the observed preservation of the architecture of the extended dimer in the wild‐type BthTx‐I and variants for all systems throughout the 1.5 μs simulation (Figures S4–S7). The average radius of gyration (*R*_*g*_) also reflects the increased conformational diversity of the extended dimer conformation with wider distributions and higher values compared to the compact conformation (Figure 3c). In particular, the average *R*_*g*_ for the wild‐type extended conformation has a multi‐modal distribution with three local maxima, indicating the presence of at least three major conformational populations. This reinforces the conclusion that the extended dimer samples multiple conformational substates, and is consistent with the proposed “hinge” hypothesis (da Silva Giotto et al. 1998). The structural reorganization at the dimer interface increases the number of contacts between the two monomers as compared to the crystal structure, and the more extensive intermolecular contacts readily explain the higher than expected values of the extended dimer stability (*D*/Δ*G*_*n*→*i*_) (see Table S1), consistent with previous guanidinium hydrochloride denaturation experiments (Ruller et al. 2003; Ruller et al. 2005). Calculation of the fractions of dimer and folded monomer from the *D*/Δ*G*_*n*→*i*_ values suggests an approximate 9:1 dimer to monomer ratio for the wild‐type and W77H/V31W proteins and a 7:3 ratio for the W77H/L10W variant at the protein concentrations used in the fluorescence experiments. It is noteworthy that despite the substantial structural differences between the compact and extended conformations, the calculated values of the average *R*_*g*_ show only slight differences, with values of 18–19 and 22–23 Å for the compact and extended dimers, respectively. Possible sample heterogeneity together with the modest difference in average *R*_*g*_ highlights the difficulties of discriminating between the two dimer conformations using techniques such as SAXS or dynamic light scattering (DLS). During submission of this report, we became aware of a recent report of MD simulations with the myotoxin II, a Lys49 sPLA_2_‐like protein from the venom of *Bothrops asper* (https://doi.org/10.1016/j.toxicon.2025.108581) with high sequence and structural similarity to the BthTx‐I, and in contrast to our experimental and MD simulations results indicates that neither the compact nor extended homodimer conformation of the protein is maintained over the time course of the simulations.

A robust metric discriminating the two dimer conformations is the average pair distance between the Trp residues (Figure 3d). In the compact conformation, the Trp‐Trp separations of 40–60 Å in the wild‐type and W77H/L10W variant fall well outside the Förster radius and preclude efficient energy transfer, whereas the W77H/V31W Trp‐Trp separation of 2–8 Å lies well within the optimal range for FRET. In sharp contrast, the Trp‐Trp separations of 40–50 Å in the W77H/V31W variant in the extended dimer conformation lie well beyond the Förster radius, whereas the Trp‐Trp separation of 5–8 Å in the wild‐type and W77H/L10W variant is fully compatible with the high energy‐transfer efficiencies experimentally observed (Figure 2a). We have also perfomed analyses of the large scale motions for the compact and extended dimer AT simulations (Figures S10 and S11) which provide further details on the molecular dynamics of these two ensembles.

**FIGURE 4:**
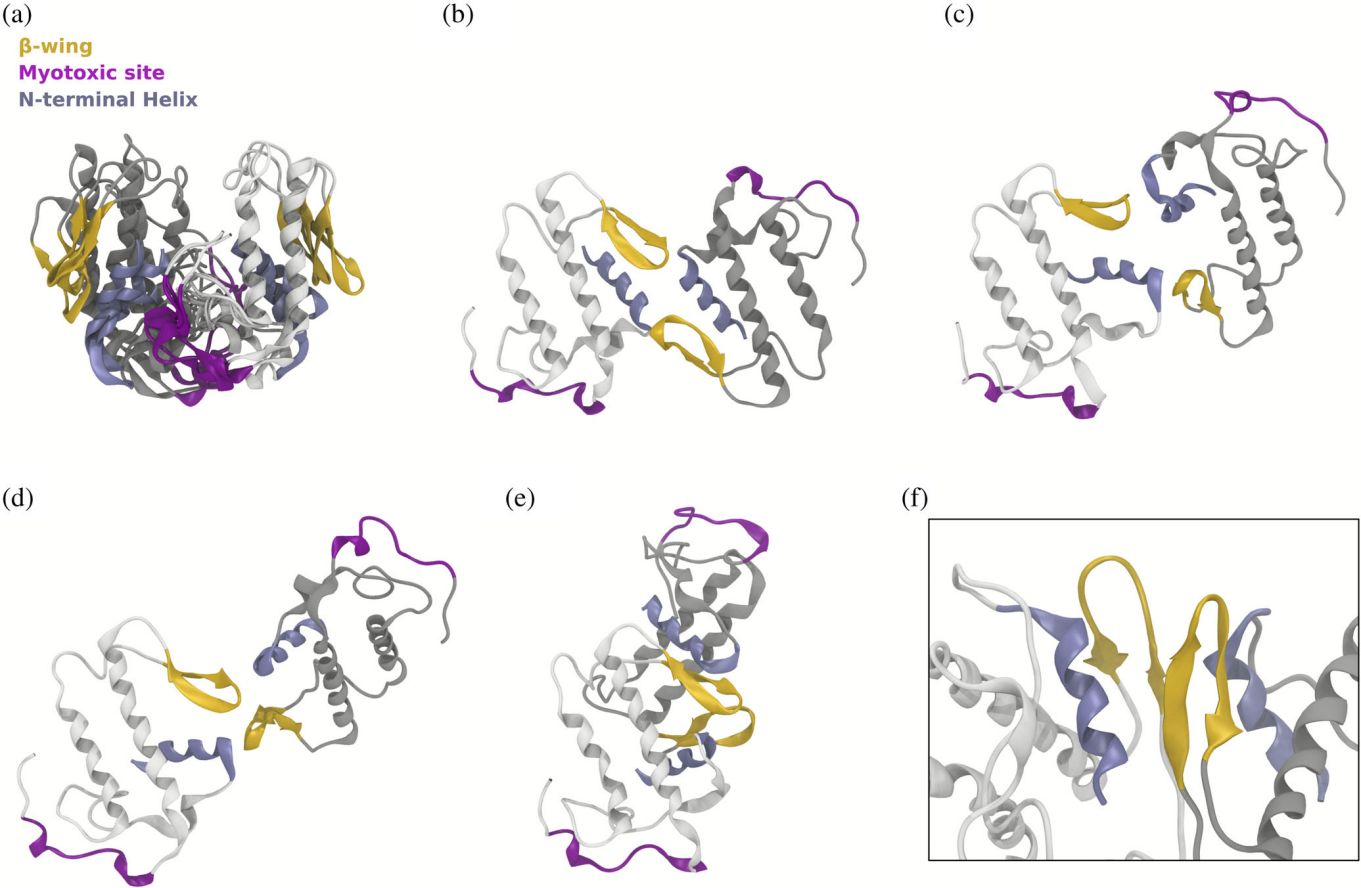
Representative conformations of the wild‐type Lys49‐PLA_2_ from the atomistic MD simulations. (a) Representative conformations taken from each independent simulation of the compact model superimposed using the alpha carbons of the larger alpha helices of monomer A (in white). The subtle alterations reflect the adaptation from the crystalline environment to solvated conditions. (b) Crystallographic structure of the extended model. (c–e) Representative conformations taken from each independent simulation of the extended dimer. (f) Detail of the interface region of the dimer shown in panel (e). The N‐terminal helix, β‐wings, and the myotoxic site are highlighted. Conformations are from the three replicas, each with 0.5 μs. Details of the structural analysis performed independently for each replica can be found in Supporting Information.

The three‐replica simulations of the compact dimer exhibit only minor structural changes from the x‐ray conformation (Figure 4a), indicating that a substantial conformational rearrangement would be required to position the C‐terminal loops of both monomers for insertion into the membrane surface. In contrast, the MD replicas of the extended conformation reveal structural ensembles that may represent successive stages along a structural transition pathway (Figure 4b–f). In the extended dimer conformation, the two monomers interact primarily through their β‐wing and N‐terminal helix regions. In the x‐ray structure, the N‐terminal helix lies parallel to the two longest helices within the same monomer and to the N‐terminal helix of the opposing monomer, while the β‐wings remain separated (Figure 4b). In solution, the transition begins with a reorientation of the monomers coupled to a displacement of the N‐terminal helix to a perpendicular orientation relative to the two longest helices of the same monomer (Figure 4c). This rearrangement brings the β‐wings into closer proximity (Figure 4d) and progresses toward a stable dimeric architecture (Figure 4e), in which the C‐terminal loops of both monomers are optimally positioned for interaction with the membrane.

Taken together, these findings establish three key points: (i) only the extended dimer geometry orients the tryptophan residues that can result in FRET efficiencies that can explain the experimental tryptophan anisotropy results of the wild‐type BthTx‐I and mutant proteins; (ii) both the compact and extended homodimer conformations observed in the crystallographic structure persist as stable quaternary structures in solution, and (iii) the compact dimer remains conformationally rigid, while the extended dimer samples multiple conformational substates which may represent intermediate structures leading to the membrane‐bound states. These findings provide a structural framework for the hypothesis that conformational variability within the dimeric assembly is functionally relevant, and enable the monomers to adapt their relative orientation upon membrane binding, thereby promoting efficient interaction with the lipid bilayer and the subsequent phospholipid membrane disruption that has been previously observed using liposomes composed of a mixture of zwitterionic and anionic phospholipids (Chioato et al. 2007; Ferreira et al. 2008). To further investigate the mechanism of membrane disruption, coarse grained molecular dynamics (CGMD) simulations of wild‐type BthTx‐I in the presence of a DPPC/DPPG membrane bilayer were performed. The CGMD simulations enable the exploration of extended timescales and large‐scale conformational transitions, providing insights to inter‐monomer rearrangements and membrane insertion events that are difficult to capture with atomistic simulations alone.

### 2.3 A molecular mechanism for PLA_2_ myotoxic activity based on conformational heterogeneity

**FIGURE 5:**
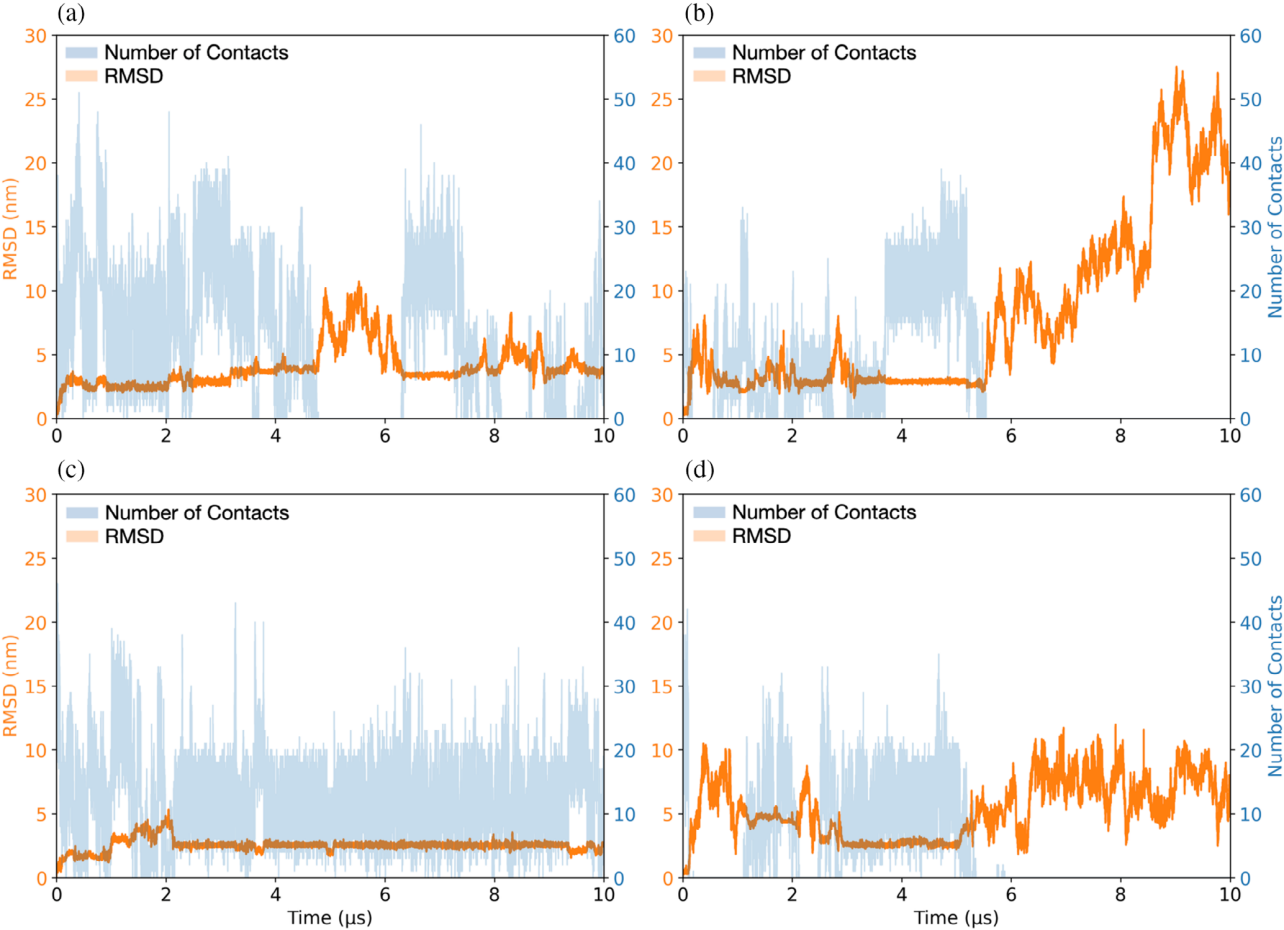
RMSD (orange) and number of contacts (blue) from the CGMD simulations for the compact homodimer configuration in (a) position 1 (P1_com_) and (b) position 2 (P2_com_), and for the extended homodimer configuration in (c) position 1 (P1_ext_) and (d) position 2 (P2_ext_). RMSD values were calculated for the given dimer by fitting the backbone beads of monomer A and then computing RMSD for the entire protein backbone. The number of contacts were calculated for the monomer backbone beads using a cutoff distance of 0.8 nm.

CGMD simulations were performed for the compact and extended dimers in two different initial positions with respect to the DPPC/DPPG membrane surface. In position 1, the i‐face of the BthTx‐I was oriented toward the solvent (systems P1_com_ and P1_ext_), and in position 2, the i‐face was oriented toward the membrane surface (systems P2_com_ and P2_ext_). The profiles of the time‐dependent RMSD and number of inter‐monomer contacts over 10 μs reveal that of the four simulated systems, the dimeric quaternary structure is maintained only in the P1_ext_ over the full course of the simulation (Figure 5c). In the remaining three systems, the dimers undergo dissociation, either reversibly as in the P1_com_ system (Figure 5a) or irreversibly in the P2_com_ and P2_ext_ systems (Figure 5b,d, respectively). The loss of inter‐monomer contacts as a result of dimer dissociation events are characterized by marked increases in RMSD values as the resulting monomers diffuse independently in the plane of the bilayer membrane. The distinct RMSD profiles and inter‐monomer contact analyses observed for the four CGMD simulations strongly suggest differences in quaternary structure stabilities of the membrane associated dimers. In order to gain insights into how membrane binding influences dimer stability, a detailed analysis of the protein–membrane contacts was performed.

**FIGURE 6:**
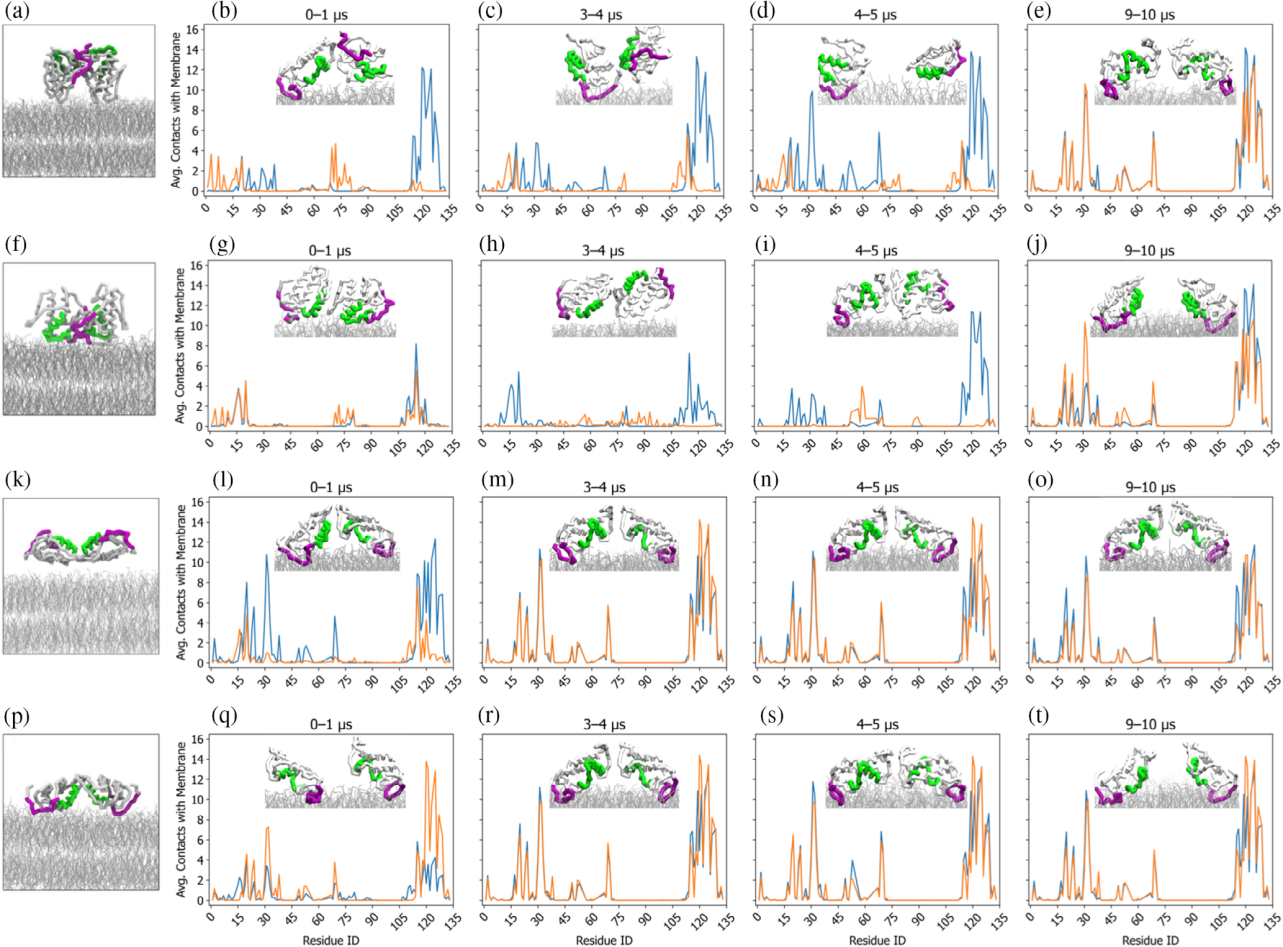
Evolution over time of protein–membrane interactions from the four CG simulated trajectories represented by average contact number over four different time intervals. The blue and orange lines represent the average contacts of Monomer A and Monomer B, respectively, with the membrane. Panels (a–e) correspond to P1_com_, (f‐Movieg) to P2_com_, (k–o) to P1_ext_, and (p–t) to P2_ext_. The initial position of each system after equilibration is illustrated in panels (a, f, k, p). Protein–membrane contacts were evaluated by measuring the distance between the center of mass of each residue and membrane atoms; contacts were defined at <8 Å, counted per frame, and averaged over the trajectory to yield per‐residue contact frequencies. For clarity, only the protein backbone is shown, with the C‐terminal loop (residues 115–129) highlighted in fuchsia and the hydrophobic channel of the i‐face (residues 1–21) shown in green. The membrane phospholipids are represented as gray lines.

The average number of protein–membrane contacts per residue for the two monomers in each homodimer conformation reveal a common trend across all four simulations (Figure 6). Early in the simulations, at least one monomer establishes extensive contacts with the membrane via the C‐terminal loop region and residues 15–40 in the hydrophobic channel (Figure 6, first column). In the extended conformations (P1_ext_ and P2_ext_), both monomers become anchored to the membrane through these regions within the first microsecond, whereas in the compact conformations (P1_com_ and P2_com_) this dual membrane anchoring is delayed and occurs only much later (~9 μs) in the simulations. These results when combined with the RMSD and inter‐monomer contact time series indicate that an adequate arrangement of the monomers in the homodimer conformation leads to a rapid membrane anchoring via amino acid residues in the C‐terminal loop and hydrophobic i‐face. The regions forming protein‐membrane contacts observed in the simulations are in broad agreement with those identified by previous experimental fluorescence data from scanning tryptophan and scanning alanine mutagenesis (Aragão et al. 2009; Chioato et al. 2007; Ferreira et al. 2008). Previous experimental investigation of the interaction of BthTx‐I with liposome membranes have demonstrated that the double mutants W77H/V31W, W77H/D67W, W77H/Y117W, and W77H/Y119W exhibit both a significant increase in tryptophan fluorescence intensity and a significant decrease in maximum emission wavelengths in the presence of phospholipids liposomes. This observation provides strong evidence that these residues are located in a more hydrophobic environment after interaction with phospholipid membranes (Ferreira et al. 2008) and are in good agreement with the CGMD simulations (Figure 6). For dimer conformations or initial positions in which contact between the C‐terminal loop/i‐face and the membrane requires substantial inter‐monomer reorientation, the structural rearrangements necessary to achieve the final membrane bound orientation leads to the disruption of the protein–protein contacts at the homodimer interface, ultimately leading to temporary or complete dimer dissociation (Figure 6). It is noteworthy that after 9 μs, the monomers in all systems converge to essentially identical protein–membrane contact profiles (Figure 6, last column), in which the i‐face is oriented toward the membrane surface and the C‐terminal loop region is inserted into the phospholipid bilayer. The maintained membrane contact of the C‐terminal loop regions of the monomers observed in the CGMD simulations suggests that the monomeric membrane bound form of the protein may be functionally relevant. The recently reported molecular dynamics simulations with the myotoxin II also included a study of the membrane bound protein (https://doi.org/10.1016/j.toxicon.2025.108581), which indicated that both the compact and extended forms dissociated into monomers, and that the membrane bound monomers display significant orientational fluctuations. Indeed, experimental modulation of the BthTx‐I dimer dissociation constant by site‐directed mutagenesis experiments of residues in the highly conserved Glu12/Trp77/Lys80 triad resulted in mutants where the monomeric form is predominant, yet even these monomeric variants present membrane permeabilization and myotoxicity activities, although at reduced levels (Ruller et al. 2005).

Only the P1_ext_ system preserves the quaternary structure conformation over the full simulation time (Figure 5c). We gained further insight into the dynamics of the membrane‐bound dimer by comparing the structures of the extended and compact conformations across all simulations using the P1_ext_ system as a reference. The resulting RMSD heatmaps comparing the P1_com_, P2_com_, and P2_ext_ trajectories to the P1_ext_ one (Figure S8a–c) reveal transient sampling of dimer conformations that are highly similar to the stable, membrane‐bound state of the P1_ext_ simulation (Figure S8d–f). This striking finding indicates that the four simulations, even if only transiently, sample high‐similarity structures (Figure S8d–f, top) which are essentially identical in overall fold but differ in membrane binding patterns (Figure S8d–f, bottom). In the P1_com_ and P2_ext_, the C‐terminal loop regions of both monomers contact the bilayer, whereas in P2_com_, one loop remains solvent‐exposed (Figure S8d–f, bottom).

We further compared the CGMD‐derived structural ensembles of the four membrane–protein systems by quantifying the quaternary structure dynamics using two geometric descriptors, the azimuthal (*θ*_A_) and tilt (*θ*_T_) angles, as previously defined (dos Santos et al. 2009) (Figure S9). The azimuthal angle (*θ*_A_) measures the relative orientation between the H3 α‐helices of each monomer and is defined by the vectors Ka and Kb, derived from the Cα atoms of Lys97 and Lys108 (Figure S9). The tilt (*θ*_T_) is defined as the angle between the planes containing the H2–H3 α‐helices of each monomer, calculated from Na = Ra × Ka and Nb = Kb × Rb, where Ra and Rb are defined by Arg43‐Lys97 (Figure S9). Using these definitions, the dimer conformations similar to the compact form have *θ*_T_ smaller than 90° and the dimer conformations similar to the extended form have *θ*_T_ greater than 90°. In the crystal structures, *θ*_A_ and *θ*_T_ are respectively 60° and 53° for the compact dimer conformation, and 148° and 122° for the extended dimer conformation.

**FIGURE 7:**
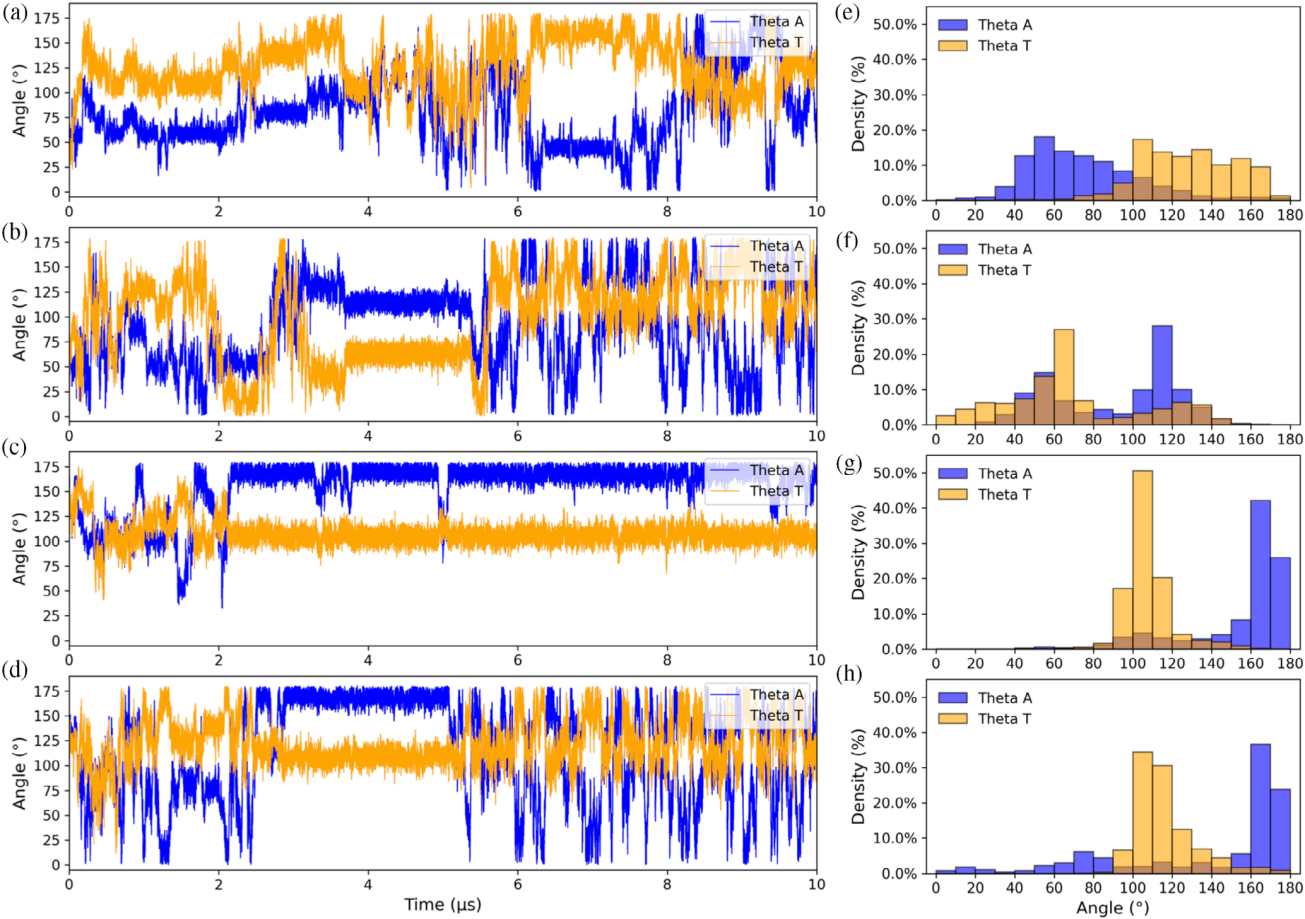
Temporal evolution of the tilt (*θ*_T_) and azimuthal (*θ*_A_) angles for CG simulations (panels a–d), together with their respective density distributions as a function of angle (panels e–h). The simulations are P1_com_ (a, e), P2_com_ (b, f), P2_ext_ (c, g), and P1_ext_ (d, h). Histograms were computed only for dimer conformations, i.e., dissociated monomers were excluded. A distance cutoff of 8.0 Å between beads in the two monomers was used to filter dimers from dissociated monomers.

In the P1_ext_ simulation, the azimuthal (*θ*_A_) and tilt (*θ*_T_) angles remain close to those observed in the crystal structure of the extended dimer conformation, supporting the presence of a stable quaternary structure in the membrane‐bound state (Figure 7a–d). In contrast, the P1_com_, P2_com_, and P2_ext_ states undergo substantial deviations in both *θ*_A_ and *θ*_T_, indicative of large‐scale inter‐monomer reorientation events during the simulations. In the P1_com_ and P2_com_ simulations, *θ*_T_ values progressively increase from compact‐like conformations (*θ*_T_ < 90°) toward extended‐like conformations (*θ*_T_ > 90°), consistent with the transitions required to reorient the C‐terminal loop regions for productive membrane binding. The variations in the corresponding values of *θ*_A_ reflect correlated twisting motions of the H3 α‐helices during this rearrangement. The angle density distributions highlight these trends (Figure 7e–h), in which P1_ext_ displays narrow distributions of *θ*_A_ and *θ*_T_ that are centered around the values observed for the extended dimer configuration observed in the crystal structures. In contrast, the P1_com_, P2_com_, and P2_ext_ systems show broad, multimodal distributions spanning both compact‐ and extended‐like conformations. These broad distributions indicate sampling of multiple quaternary arrangements and are consistent with the transient adoption, but poor stabilization of the membrane binding geometry.

In summary, our results from atomistic molecular dynamics simulations indicate that both the compact and extended quaternary arrangements of the BthTx‐I dimer are structurally feasible and may co‐exist in aqueous solution. However, experimental evidence from fluorescence anisotropy data strongly suggests the presence of the extended dimer configuration at protein concentrations used in myotoxic activity assays in vivo. CGMD simulations indicate that protein‐phospholipid contacts via the C‐terminal loop and i‐face surface are the primary driver of inter‐monomer reorientation within the membrane‐bound dimer. In those cases where this reorientation is significant, such as assemblies starting from less favorable geometries, the structural adjustments required to position the C‐terminal loop toward the membrane destabilize the quaternary interface that ultimately leads to partial or complete dimer dissociation. Angular analyses of the inter‐monomer dynamics confirm that these rearrangements coincide with the loss of dimer interface stability. This coupling between quaternary stability and membrane‐binding geometry explains why specific arrangements, exemplified by the P1_ext_ conformation, favor the productive orientation needed for simultaneous anchoring of both C‐terminal loops, enabling efficient membrane insertion and phospholipid disruption central to the myotoxic mechanism of the Lys49 sPLA_2_‐like proteins. Our results identify a functionally competent membrane‐bound conformation and also reveal that the dimer free in solution samples a broad conformational landscape, encompassing structural states consistent with both crystallographic dimer arrangements observed in the asymmetric unit. This structural plasticity may represent an inherent property of the sPLA_2_‐like proteins, enabling functional adaptability while posing challenges for experimental characterization.

### 2.4 Critical assessment of available experimental SAXS data for determination of dimer conformation

The suggestion that Lys49 PLA_2_‐like proteins adopt a compact dimeric conformation in solution (Borges et al. 2017) was originally based on SAXS data (Murakami et al. 2007). It is noteworthy that these data were acquired and analyzed without the explicit assessment of polydispersity or conformational heterogeneity. Under these conditions, SAXS provides only intensity‐averaged profiles, and individual conformations cannot be resolved without ensemble refinement. The compact dimer conformation was suggested on the basis of subtle differences near the intrinsic resolution limit of the method (Δ*R*_*g*_ of only a few Å, NSD values of 1.21 vs. 1.85), which are insufficient to exclude coexisting quaternary states. The assessment of polydispersity or conformational heterogeneity has since emerged as standard for oligomeric and flexible proteins (Kikhney and Svergun 2015; Korasick and Tanner 2018; Yang 2014), and more recent SAXS studies have accounted for sample heterogeneity, reporting *R*_*g*_ values of 18.0 ± 0.2 Å for monomer‐rich samples and 34.8–39.2 Å for tetramer‐dominated mixtures. Nevertheless, these studies could not isolate a pure dimer population (Cardoso et al. 2021) and the samples displayed high polydispersity (~20–30%), well above the <10% expected for monodisperse proteins. These studies further indicate that the reported *R*_*g*_ values represent population averages rather than discrete oligomeric states and highlight the challenges of using SAXS for distinguishing alternative quaternary arrangements in small proteins such as sPLA_2_s (~26 kDa). We have therefore performed theoretical calculations in order to gain a notion of the limits of SAXS experiments.

**FIGURE 8:**
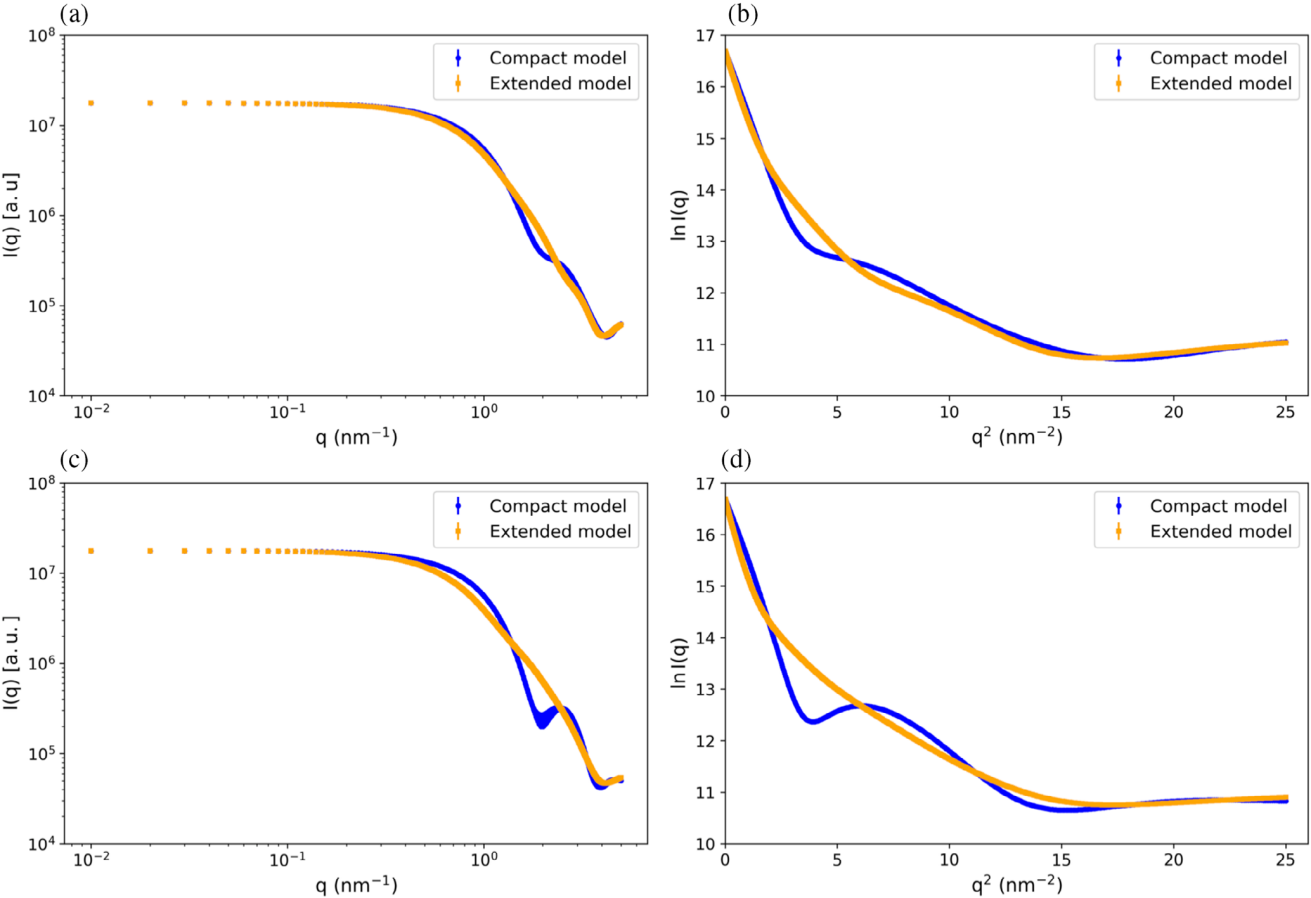
Theoretical SAXS profiles for BthTx‐I dimers. SAXS intensity curves for (a) a single X‐ray conformation of the compact and extended dimer conformations and (c) multiple conformations sampled over the last 50 ns of ATMD simulations starting from each assembly, respectively. Corresponding Guinier plots for the (b) X‐ray single conformation and (d) structural ensembles from the CGMD simulations.

Profiles derived from the x‐ray structures of the compact and extended BthTx‐I dimer conformations (Figure 8a,b), as well as from structural ensembles sampled during the final 1 μs of their respective CGMD simulations (Figure 8c,d), yielded nearly overlapping curves. At low‐q, Guinier analyses show slopes differing by only ~1 Å in *R*_*g*_, well below the experimental resolution for proteins of this size. In the intermediate‐q region, which encodes overall shape and relative domain orientation, the differences between the assemblies are more representative and are further increased by conformational averaging in the MD ensembles. At high‐q, dominated by local packing, the curves are very similar. Consistently, *χ* ^2^ values between the two calculated profiles are <10^−3^, confirming that despite their atomic‐level differences, the scattering signatures of the two dimer conformations fall within the experimental uncertainty of SAXS. These theoretical considerations highlight the ambiguities inherent in the interpretation of SAXS data for discrimination between alternative dimer conformations of the Lys49 PLA_2_‐like proteins.

## 3 MATERIALS AND METHODS

### 3.1 Site‐directed mutagenesis and recombinant protein expression and purification

The wild‐type Bothropstoxin‐I (BthTx‐I) contains a single tryptophan residue at position 77 (W77), and the substitution by histidine (W77H) has no significant effects on the membrane damaging and myotoxic activities (Ruller et al. 2005). Additional substitutions with tryptophan residues by site‐directed mutagenesis using the W77H as a template can be used to generate variants of the BthTx‐I with single tryptophan residues at the proposed homodimer interfaces in both the compact (W77H/V31W) and extended conformations (W77H/L10W) (see Figure 1). The site‐directed mutagenesis to generate the double mutants W77H/L10W and W77H/V31W has been previously described (Ferreira et al. 2008). The expression of recombinant BthTx‐I and variants in *Escherichia coli*, refolding from inclusion bodies and subsequent purification by cation‐exchange chromatography was performed as previously reported (Ward et al. 2001). Protein purity was routinely evaluated by silver staining of SDS‐PAGE (Laemmli 1970), and aliquots of purified protein were stored at 4°C for no longer than 1 month prior to use in experiments.

### 3.2 Tryptophan emission anisotropy measurements

Samples of recombinant BthTx‐I and mutants at a final concentration of 30 μg mL^−1^ were prepared in a HEPES buffer (20 mM de HEPES, 150 mM de NaCl, pH 7.0). For equilibrium denaturation experiments, the guanidinium hydrochloride (GdnHCl) concentration in individual protein samples was increased over the range 0–8M by dilution of a filtered 10M stock solution of denaturant prepared in the same buffer. All samples were incubated for 12 h at 298 K prior to the spectroscopic measurements. Steady state intrinsic tryptophan emission anisotropy measurements were performed in 1 cm optical path length quartz cuvettes using a SLM‐AMINCO 8100 fluorescence spectrometer (SLM, Urbana, IL) operating in a “L‐format” with excitation and emission slit widths of 4 nm and a photomultiplier tube voltage of 600 V. The fluorescence intensities of the vertical and horizontal emission components on excitation with vertically polarized light (*I*_vv_ and *I*_vh_) were measured at a fixed emission wavelength of 350 nm on excitation of the sample at 295 nm. The anisotropy signals from buffer blanks were subtracted, and the polarization (*P*) of the emission was calculated using

(1) $$P=\frac{I_{\mathrm{vv}}-GI_{\mathrm{vh}}}{I_{\mathrm{vv}}+GI_{\mathrm{vh}}},$$

where *G* was a machine‐dependent correction factor measured with horizontally polarized excitation. From this value of *P*, the emission anisotropy, $\left\langle r\right\rangle$, was calculated according to

(2) $$\left\langle r\right\rangle =\frac{2P}{3-P}.$$

Measurements were performed at 298 K, and all protein emission spectra were corrected by subtraction of the spectrum from the equivalent buffer at the given GdnHCl concentration.

The steady state intrinsic tryptophan emission anisotropy data were analyzed using a three‐state denaturation model as previously described (Ruller et al. 2003), which may be summarized as

(3) $$N_{2}↔2M↔2U,$$

in which the dimeric native form of the protein (*N*_2_) is assumed to dissociate into a folded monomeric state (*M*) which subsequently denatures to yield the unfolded monomeric form (*U*) of the protein. Mathematical functions describing such a three‐state model have been previously described (Park and Bedouelle 1998) and were used to fit the anisotropy data with a non‐linear least square algorithm implemented in Origin 8.5 (Microcal Software, Inc.). Single exponential functions were used for the pre‐ and post‐transition baselines in the non‐linear least squares fitting program as previously reported (Ruller et al. 2005). The least square fitting analysis yielded estimates of the Gibbs free energy of denaturation (Δ*G*) and cooperativity (*m*) for the *N*_2_ → 2*M* (*D*/Δ*G*_*n*→*m*_ and *m*_*n*→*m*_ in Table S1) and 2*M* → 2*U* (*D*/Δ*G*_*m*→*u*_ and *m*_*m*→*u*_ in Table S1) transitions.

### 3.3 Atomistic MD simulations

Atomistic MD simulations were performed for BthTx‐I in both the compact and extended dimeric conformations (Figure 1 and Table 1). The initial atomic coordinates for the wild‐type (WT) protein in both dimeric arrangements were obtained from the crystal structure of the BthTx‐I (RCSB PDB database ID: 3HZD) (Fernandes et al. 2010; Silva et al. 2009). The atomic coordinates for the two homodimer conformations were selected from the positions of the protein molecules in the asymmetric unit in the PDB file and are available at https://github.com/BioMat-USP-RP/Simulations-of-Lys49-PLA2-homologues. Both dimer conformations were simulated for the wild‐type sequence and two double mutants: L10W/W77H and V31W/W77H, where the mutant coordinates were generated with PyMol (Schrödinger LLC 2015). Each protein was placed in a cubic box using a minimum solute‐wall distance of 3.0 nm. In order to remove possible strains caused by the difference between the force‐field parameters and the crystallographic structure, each protein structure initially underwent unconstrained energy minimization in vacuum using the steep descent algorithm until convergence with a minimization step size of 0.01 kJ mol^−1^ and a force tolerance of 10.0 kJ mol^−1^ nm^−1^. The systems were solvated using the simple point charge (SPC) water model (Berendsen et al. 1981). A total of 22 chloride ions were added to each system, which is sufficient to neutralize the protein charge. To relax unfavorable contacts between protein atoms, ions and the solvent, a second energy minimization, identical to the first, was performed. The equilibration protocol consisted of five 100 ps simulations where the protein atoms were harmonically restrained to their initial positions. The first simulation was performed in the NVT ensemble with a force constant of 200 kJ mol^−1^ nm^−2^ for positional restraints. Initial atomic velocities were sampled from a Maxwell distribution at 300 K. The remaining four stages of the equilibration were performed in the NPT ensemble with force constants of 200, 150, 100, and 50 kJ mol^−1^ nm^−2^ consecutively. The resulting atomic coordinates were used as the initial configurations for the 500 ns production MD in the NPT ensemble. Three independent simulations, with distinct initial atomic velocities, were performed for each system. All simulations were performed at 300 K using a velocity‐rescaling thermostat with a relaxation constant of 0.1 ps (Bussi et al. 2007). The temperature of water with counter‐ions and protein were coupled separately by the thermostat. The pressure was maintained at 1 bar by the Parrinello‐Rahman barostat with a coupling time constant of 2 ps. Isotropic pressure coupling was applied with an isothermal compressibility of 4.5 × 10^−5^ bar^−1^ (Parrinello and Rahman 1981). All systems were simulated using the leap‐frog algorithm with a time step of 2 fs (Hockney et al. 1974). The center of mass motion of the system was removed every 100 time steps (0.2 ps). Energies and atomic coordinates were stored every 3000 time steps (6 ps). Hydrogen bond lengths within the solute and the geometry of water molecules were constrained using LINCS and SETTLE algorithms, respectively (Hess et al. 1997; Miyamoto and Kollman 1992). The GROMOS parameter set 54a7 was used for protein and water molecules (Schmid et al. 2011), and the 53a6 parameter set was used to describe the ions (Oostenbrink et al. 2004; Oostenbrink et al. 2005). Electrostatic interactions were calculated using the smooth particle mesh Ewald (SPME) method with cubic interpolation of charges on a Fourier grid with 0.16 nm spacing (Essmann et al. 1995). The neighbor lists were updated every 10 steps using the Verlet algorithm with a cut‐off 1.2 nm and a buffer tolerance of 0.005 kJ mol^−1^ ps^−1^. VdW interactions were modeled by the Lennard‐Jones potential shifted to be zero at the cut‐off distance. Periodic boundary conditions were applied in all directions. GROMOS 54a7 was originally parameterized using a twin‐range cutoff scheme. When applied with modern PME‐based single‐range schemes in GROMACS, small deviations in bulk properties such as density may occur. In this study, we verified that these effects do not influence the structural and dynamical observables of interest. All simulations were performed using the 2021.3 version of GROMACS (Abraham et al. 2015; Lindahl and der Hess 2021). Renders of protein crystal structures and simulation snapshots were generated using VMD (Humphrey et al. 1996; Stone 1998). All graphs in this work were generated with MatplotLib (Hunter 2007).

**TABLE 1:** Description of all simulated systems.

Systems	Number of components						Resolution	Dimer model	Total sim. time (μs)
	Dimer	POPC	POPG	Water	Na^+^	Cl^−^			
WT	1	‐	‐	65,326	‐	22	AT	Compact	1.5
W77H/L10W	1	‐	‐	67,808	‐	22	AT	Compact	1.5
W77H/V31W	1	‐	‐	67,822	‐	22	AT	Compact	1.5
WT	1	‐	‐	80,020	‐	22	AT	Extended	1.5
W77H/L10W	1	‐	‐	79,960	‐	22	AT	Extended	1.5
W77H/V31W	1	‐	‐	79,960	‐	22	AT	Extended	1.5
P1_com_	1	1000	1000	72,808	978	‐	CG	Compact	10
P1_ext_	1	1000	1000	69,804	978	‐	CG	Extended	10
P2_com_	1	1000	1000	72,803	978	‐	CG	Compact	10
P2_ext_	1	1000	1000	69,814	978	‐	CG	Extended	10

*Note*: WT indicates the wild‐type. In W77H/L10W, residue L10 was mutated to W, and in W77H/V31L, residues W77 and V31 were mutated to H and L, respectively, in the wild‐type sequence. In P1 the protein is oriented toward the solvent, whereas in P2 it is oriented toward the membrane. *Com* and *ext* describe the dimeric configurations derived from WT, which means compact and extended, respectively. AT represents atomistic models, and CG represents coarse‐grained models.

### 3.4 Coarse‐grained MD simulations

Coarse‐grained (CG) MD simulations were performed for compact and extended homodimer conformations in the presence of a bilayer composed of the phospholipids 1,2‐Dipalmitoyl‐sn‐glycero‐3‐phosphocholine (DPPC) and 1,2‐Dipalmitoyl‐sn‐glycero‐3‐phosphoglycerol (DPPG) in a 1:1 ratio (Table 1). The protein structures were mapped into the CG representation using the atomic coordinates from the PDB structure 3HZD (Fernandes et al. 2010; Silva et al. 2009). The CG coordinates and the corresponding topologies were generated using the Martinize 2.5 Python script (de Jong et al. 2013). For the phospholipid bilayer, the setup was built using the MARTINI 3 force field, with initial coordinates generated by the CHARMM‐GUI website (Alessandri et al. 2022; Marrink et al. 2007; Qi et al. 2015). Four membrane–protein CG systems were constructed using either the compact or extended homodimers, with each protein assembly in two possible orientations relative to the DPPC/DPPG membrane bilayer (Table 1). In position 1, the interface recognition site (i‐face) was oriented toward the solvent (systems P1_com_ and P1_ext_), and in position 2, the i‐face was oriented toward the membrane surface (systems P2_com_ and P2_ext_). All systems were solvated using the MARTINI CG regular water model with each bead representing four water molecules (Souza et al. 2021). 978 sodium ions were added to neutralize the total charge of the system. To prevent major strains in the initial configuration from disturbing the initial orientation of the protein in the membrane, the systems were carefully equilibrated. The geometry of the systems was optimized for 10,000 steps using the Steepest Descent algorithm and equilibrated in the NPT ensemble with the progressive increase of the time step from 0.002 ps to 0.020 ps. Each incremental time step was conducted for ~1 ns. Lipid heads and protein beads were harmonically restrained to their initial positions. In each successive simulation, the force constant of the harmonic potential was incrementally decreased as the time step was increased. During the equilibration step, the Berendsen barostat was used with a semi‐isotropic pressure coupling of 5.0 ps (Berendsen et al. 1984), whereas in the production phase the Parrinello‐Rahman barostat (Parrinello and Rahman 1981) was applied with the pressure coupling of 12.0 ps. Throughout the simulation, the reference pressure was kept at 1.0 bar and the isothermal compressibility at 3 × 10^−4^ (kJ mol^−1^ nm^−3^)^−1^. A velocity‐rescaling algorithm was employed for temperature control with a coupling time constant of 1.0 ps at 310.15 K (Bussi et al. 2007). The neighbor lists were constructed using the Verlet algorithm with a buffer tolerance of 0.005 kJ mol^−1^ ps^−1^ (Qi et al. 2015). Electrostatic interactions were treated using the reaction‐field method with a relative dielectric constant of 15 and a cutoff of 1.1 nm, following MARTINI 3 recommendations (de Jong et al. 2016; Souza et al. 2021; Tironi et al. 1995). Van der Waals interactions were treated through the Lennard‐Jones potential shifted to zero at the cut‐off distance of 1.1 nm. Periodic boundary conditions were applied in all directions. MD simulations were performed with the GROMACS software v. 2021.3 (Abraham et al. 2015; Lindahl and der Hess 2021). Trajectories were written at 1000‐step intervals.

### 3.5 Calculation of FRET efficiency from simulation

FRET rates strongly depend on the distance separating donor and acceptor fluorophores (*R*) and the orientation factor between the fluorophore transition dipoles ($\kappa^{2}$) defined as

(4) $$\kappa^{2}={\left(\cos\theta_{t}-3\cos\theta_{d}\cos\theta_{a}\right)}^{2}.$$

In the present study, $\theta_{t}$ is the angle between the transition dipoles of the donor and acceptor tryptophans (Trp), $\theta_{d}$ is the angle between the transition dipole of the donor Trp and the vector separating the center of mass of the side chains groups of donor and acceptor, $\theta_{a}$ is the angle between the same separation vector and the acceptor Trp transition dipole (Figure S1a). While the absorption and emission spectra of Trp involve two transition states, designated ^1^L_a_ and ^1^L_b_, the contribution of the latter to the emission spectra is expected to be small (Daura et al. 1999; Valeur and Weber 1977), and the analysis therefore focused on the ^1^L_a_ state. The transition dipole moment of the ^1^L_a_ state was defined by taking the main axis of the Trp side chain and rotating it by an angle of −38° in the plane of the indole ring (Figure S1b) (Yamamoto and Tanaka 1972). From the values of $\kappa^{2}$ is possible to calculate the Förster critical distance $R_{0}$, which is the distance where the energy transfer efficiency between the Trp residues is 50%,

(5) $$R_{0}=9.78\times 10^{3}\;{\left(\kappa^{2}n^{-4}J\left(\lambda \right)Q_{D}\right)}^{1/6}.$$

Here, *n* is the refractive index of the medium (assumed to be 1.4 for water), $Q_{D}$ is the quantum yield of the donor Trp residue, and $J\left(\lambda \right)$ is the overlap integral of donor and acceptor. $Q_{D}$ and $J\left(\lambda \right)$ were obtained experimentally (Table S2), while $\kappa^{2}$ was calculated from the atomistic simulations (Figure S2). Using the values of R obtained from simulations (Figure S2) in conjunction with $R_{0}$ (Figure S3), the energy transfer efficiency (*E*) was calculated,

(6) $$E=1/(1+{\left(\frac{R}{R_{0}}\right)}^{6}).$$

For each step of the simulations the values of $\kappa^{2}$ and $R_{0}$ were calculated, thereby generating the distributions of E for all conformations of both the compact and extended homodimer conformations of the wild‐type and the W77H/L10W and W77H/V31W variants (Figure 1).

## 4 CONCLUSIONS

Our integrated experimental and computational analyses reveal that both compact and extended quaternary arrangements of the BthTx‐I dimer are structurally feasible and can coexist in solution. Fluorescence anisotropy measurements strongly favor the extended conformation as the predominant species under physiologically relevant conditions. Atomistic MD simulations demonstrate that while the compact dimer is conformationally rigid, the extended dimer samples multiple substates consistent with a hinge‐like mechanism that could facilitate membrane engagement. Coarse‐grained MD simulations further establish that membrane anchoring is driven by interactions of the C‐terminal loop and hydrophobic i‐face with the bilayer. Productive and simultaneous insertion of both C‐terminal loops is achieved only when the dimer adopts a geometry similar to the extended P1_ext_ arrangement, whereas unfavorable starting orientations require substantial inter‐monomer reorientation, often leading to partial or complete dimer dissociation.

Together, these results support a mechanistic model in which conformational heterogeneity is not a mere artifact but a functional feature that enables the dimer to adapt its quaternary arrangement for productive membrane engagement and subsequent phospholipid disruption. This structural plasticity provides a unifying explanation for the diverse crystallographic arrangements observed for Lys49 sPLA_2_‐like proteins and establishes a framework for rational design of inhibitors targeting their myotoxic activity.

## AUTHOR CONTRIBUTIONS

**Diane C. A. Lima:** Investigation; validation; formal analysis; writing – original draft. **Vinicius Firmino dos Santos:** Investigation; validation; formal analysis; writing – original draft; supervision. **Bernardo Rassi:** Investigation. **Richard J. Ward:** Conceptualization; investigation; writing – original draft; writing – review and editing. **Thereza A. Soares:** Conceptualization; methodology; writing – original draft; writing – review and editing; supervision; project administration; funding acquisition.

## CONFLICT OF INTEREST STATEMENT

The authors declare no conflicts of interest.

## Supporting information


**Data S1.** Supporting Information. Protein thermodynamic stabilities from GdnHCl denaturation experiments, experimental values for quantum yield ($Q_{D}$) and spectral overlap integral ($J\left(\lambda \right)$) for the wild‐type BthTx‐I and mutants. Definition of the vector describing the indole transition dipole moment. Scatter plots for the orientation factor (*k* ^2^), time series for the average properties presented in the manuscript and room‐mean‐square deviation heat maps. Representation of the vectors used to calculate azimuth (*θ*_A_) and tile (*θ*_T_) angles.
**Figure S10.** (A) Eigenvalues and the two first eigenvectors representative of most large amplitude motions sampled through the AT simulations of (B) Assembly 1 and (C) Assembly 2. Assembly 2 displays higher eigenvalues for the first few principal components; indicative of larger amplitude collective motions compared to Assembly 1. The latter shows a faster decay of eigenvalues, reflecting more restricted large‐scale dynamics consistent with the rigid conformational ensemble observed in the AT MD simulations.
**Figure S11.** Animations of the projection of the molecular‐dynamics trajectory onto the two largest eigenvectors obtained from covariance‐matrix diagonalization of atomic positional fluctuations for Assembly 1 and Assembly 2. The animation illustrates the large‐scale collective motion associated with the two largest eigenvalue, shown by displacements along the positive and negative directions of the eigenvector relative to the mean structure.

## Data Availability

The structures, atomic parameters and input files used in the simulations, final structures obtained in the simulations, scripts used for analysis and the raw data that support the findings of this study are openly available in our public GitHub repository at https://github.com/BioMat-USP-RP/Simulations-of-Lys49-PLA2-homologues.
