# Supplementary material for: The many dimeric faces of Lys49 PLA2‐like proteins: Conformational plasticity and membrane binding drive functional dimer states
Source: Protein Sci. 2026 Jan 20;35(2):e70449. doi: 10.1002/pro.70449 (PMC12817292; doi:10.1002/pro.70449)
Supplement: Supplementary file 1 — Data S1. Supporting Information. Protein thermodynamic stabilities from GdnHCl denaturation experiments, experimental values for quantum yield (QD) and spectral overlap integral (Jλ) for the wild‐type BthTx‐I and mutants. Definition of the vector describing the indole transition dipole moment. Scatter plots for the orientation factor (k 2), time series for the average properties presented in the manuscript and room‐mean‐square deviation heat maps. Representation of the vectors used to calculate azimuth (θ A) and tile (θ T) angles. Figure S10. (A) Eigenvalues and the two first eigenvectors representative of most large amplitude motions sampled through the AT simulations of (B) Assembly 1 and (C) Assembly 2. Assembly 2 displays higher eigenvalues for the first few principal components; indicative of larger amplitude collective motions compared to Assembly 1. The latter shows a faster decay of eigenvalues, reflecting more restricted large‐scale dynamics consistent with the rigid conformational ensemble observed in the AT MD simulations. Figure S11. Animations of the projection of the molecular‐dynamics trajectory onto the two largest eigenvectors obtained from covariance‐matrix diagonalization of atomic positional fluctuations for Assembly 1 and Assembly 2. The animation illustrates the large‐scale collective motion associated with the two largest eigenvalue, shown by displacements along the positive and negative directions of the eigenvector relative to the mean structure. [file PRO-35-e70449-s001.zip › Revised-Supplementary-Information-2025.10.25.docx]

**THE MANY DIMERIC FACES OF LYS49 PLA₂-LIKE PROTEINS: CONFORMATIONAL PLASTICITY AND MEMBRANE BINDING DRIVE FUNCTIONAL DIMER STATES**

*Diane C. A. Lima,^1,#^ Vinicius Firmino dos Santos,^1,#^ Bernardo Rassi,^1^*

*Richard J. Ward,^1*^ Thereza A. Soares.^1,2*^*

^1^Department of Chemistry, University of São Paulo, 14040-901 Ribeirão Preto, Brazil.

^2^Hylleraas Centre for Quantum Molecular Sciences, University of Oslo, 0315 Oslo, Norway.

*Corresponding Author: [rjward@ffclrp.usp.br](mailto:rjward@ffclrp.usp.br), [Thereza A. Soares](mailto:thereza.soares@usp.br)

#These authors contributed equally to this work.

Running Title: Dimer Plasticity and Membrane Binding in Lys49 sPLA₂s

**Table S1.** Thermodynamic stabilities of the dimeric and monomeric BthTx-I as estimated from GdnHCl denaturation experiments.

| **Protein** | **r_n_** | **r_i_** | **r_u_** | **D/greek/G_n→i_** | **m_n→i_** | **D/greek/G_i→u_** | **m_i→u_** |
| --- | --- | --- | --- | --- | --- | --- | --- |
| WT | 0.084 | 0.10 | 0.066 | 9.8 | 1.4 | 7.2 | 1.8 |
| W77H/L10W | 0.086 | 0.10 | 0.044 | 8.5 | 1.5 | 6.4 | 2.0 |
| W77H/V31W | 0.13 | 0.11 | 0.078 | 10.0 | 0.9 | 7.2 | 2.1 |

**Table S2.** Experimental values for quantum yield ($Q_{D}$) and spectral overlap integral ($J(\lambda)$) for the wild-type BthTx-I and mutants.

| **Protein sequence** | ***Q_D_*** | ***J(𝜆)* (M^-1^cm^-3^)** |
| --- | --- | --- |
| Wild-type (W77) | 0.14 | 4.20×10^-17^ |
| W77H/L10W | 0.18 | 5.16×10^-18^ |
| W77H/V31W | 0.13 | 1.87×10^-17^ |


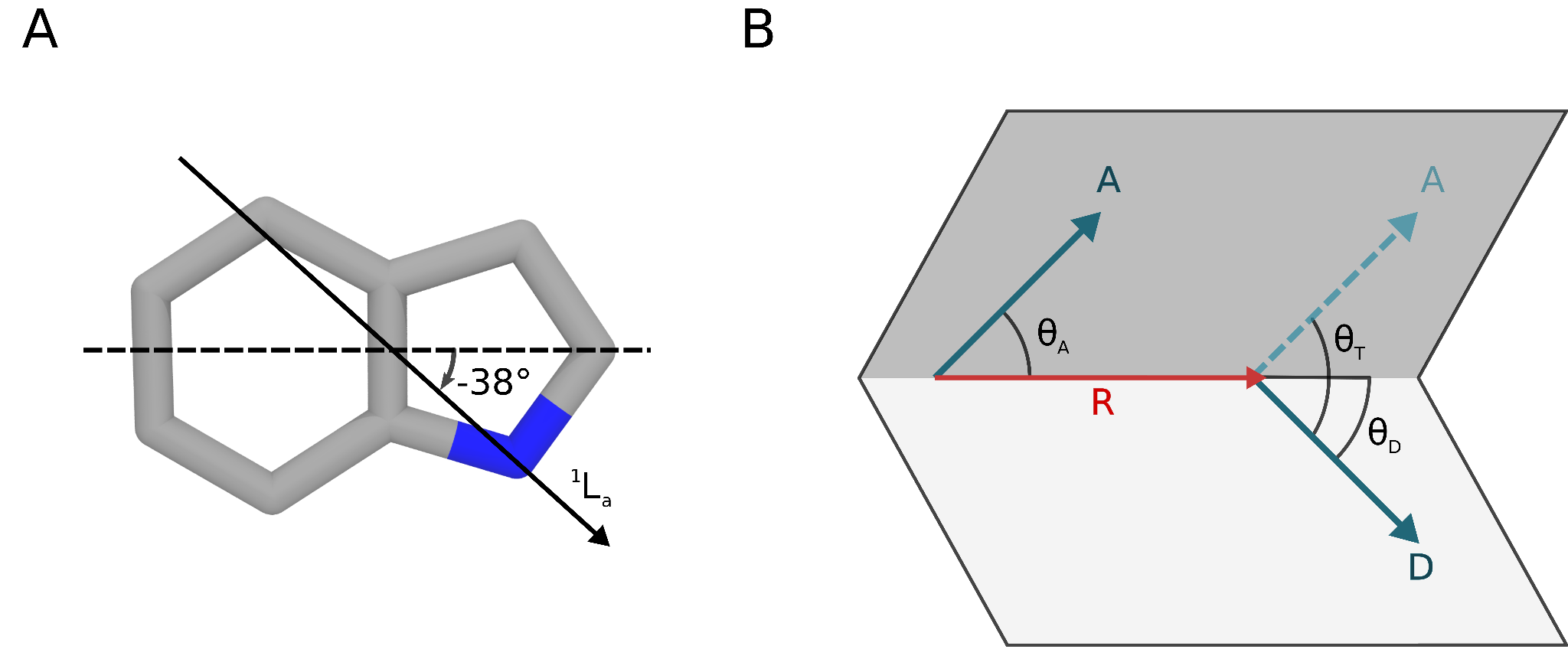


**Figure S1.** **A)** Definition of the vector describing the indole transition dipole moment ^1^L_a_ of the tryptophan residue side chain **B)** Definition of angles between acceptor (A) and donor (D) transition dipole moments and separation vector (R) used in the calculation of the orientation factor ($\kappa^{2}$).

#

# **
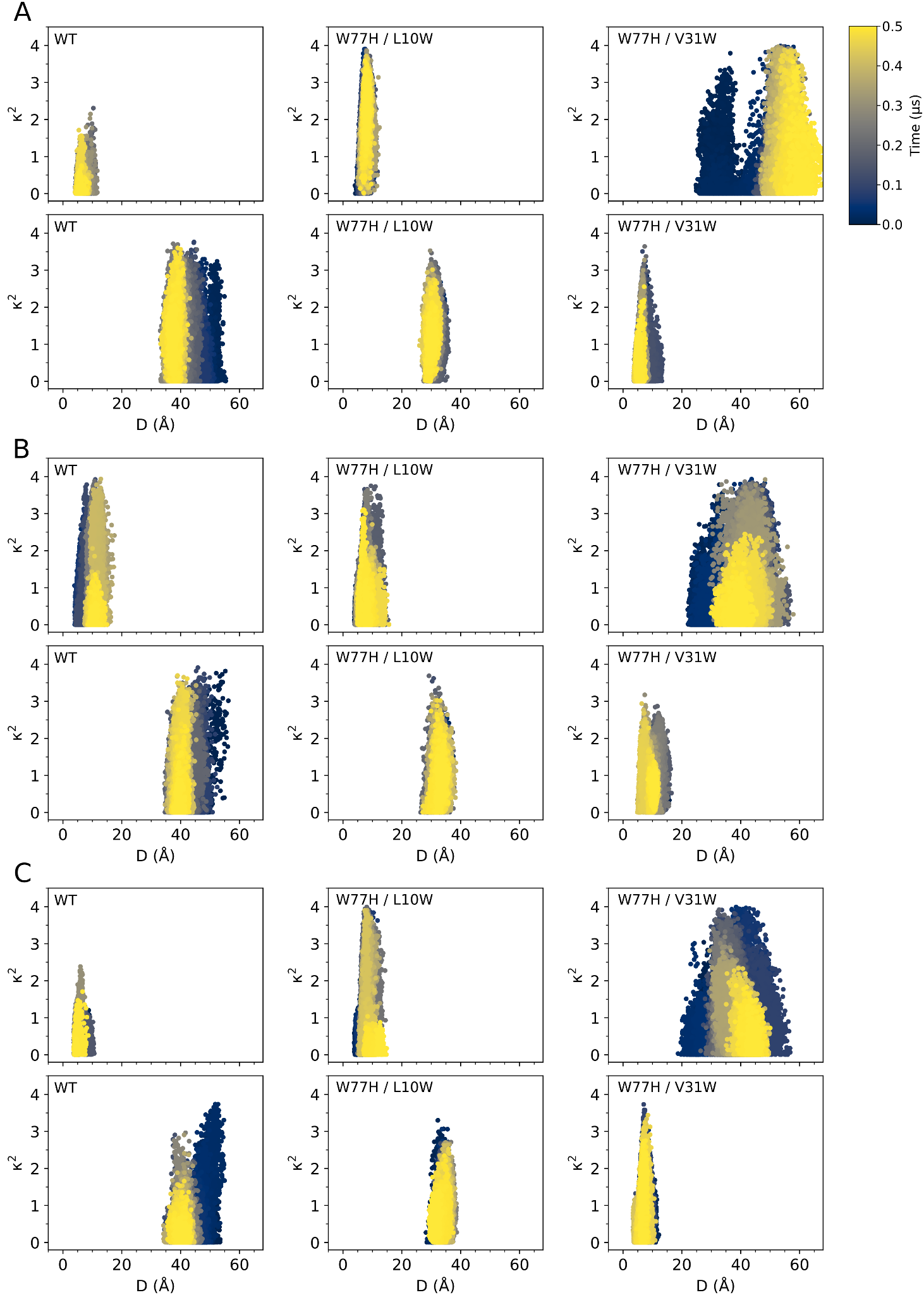
**

# **Figure S2.** Scatter plots for the orientation factor (k^2^) as a function of the distance (D) between the centers-of-geometry of the donor and acceptor indole groups for three independent simulations (**A, B, C**) of the Compact (upper rows of panels) and Extended (lower rows of panels) BthTx-I homodimer models. The coloring scheme represents simulation time, with blue at the beginning of the simulation and yellow at the end.

#
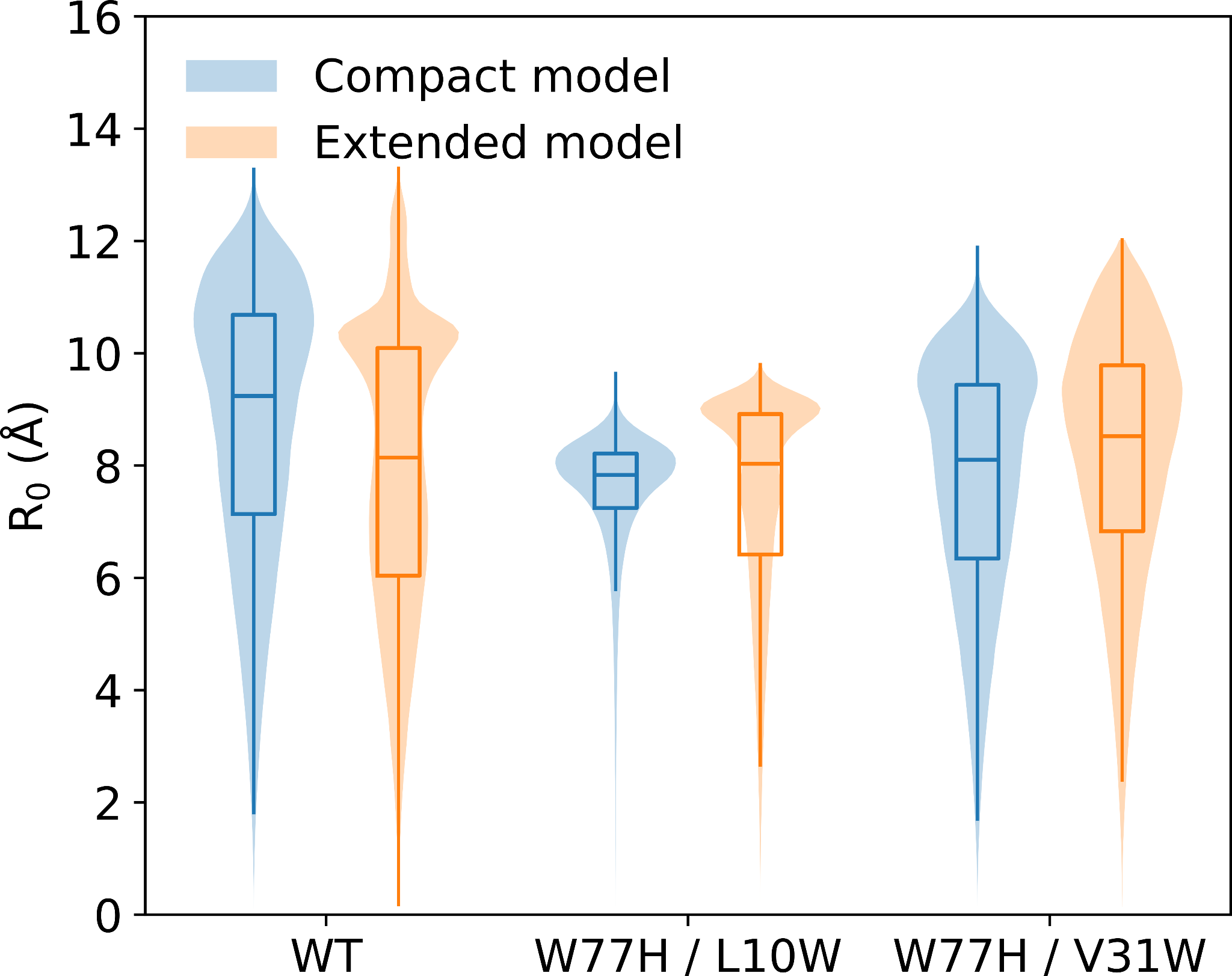


**Figure S3**. Förster radius (critical transfer distances, R_0_) calculated from simulations of the Compact (light blue) and Extended (light orange) BthTx-I homodimer models. Statistics were calculated for over three independent simulations of each protein, where the entire production MD (for a total of 1.5 µs for all three replicas) was used.

**
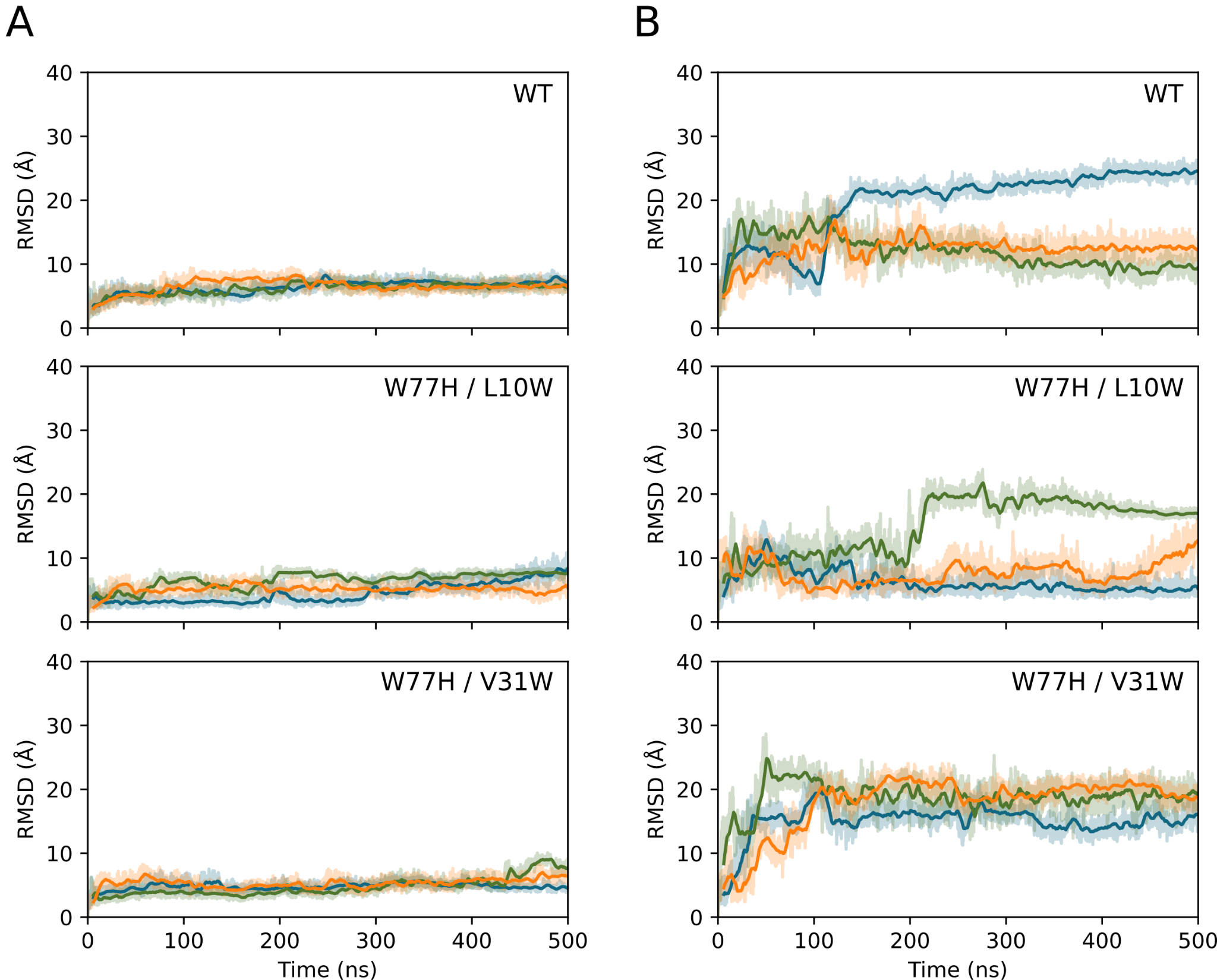
**

**Figure S4.** Time-series for the root mean square deviations (RMSD) for simulations of the Compact (**A**) and Extended (**B**) homodimer models of the wild-type (WT), together with the W77H/L10W and W77H/V31W mutants (as indicated the figure panels). The results from the three replicate simulations for each protein are presented in shades of blue, orange and green.

**
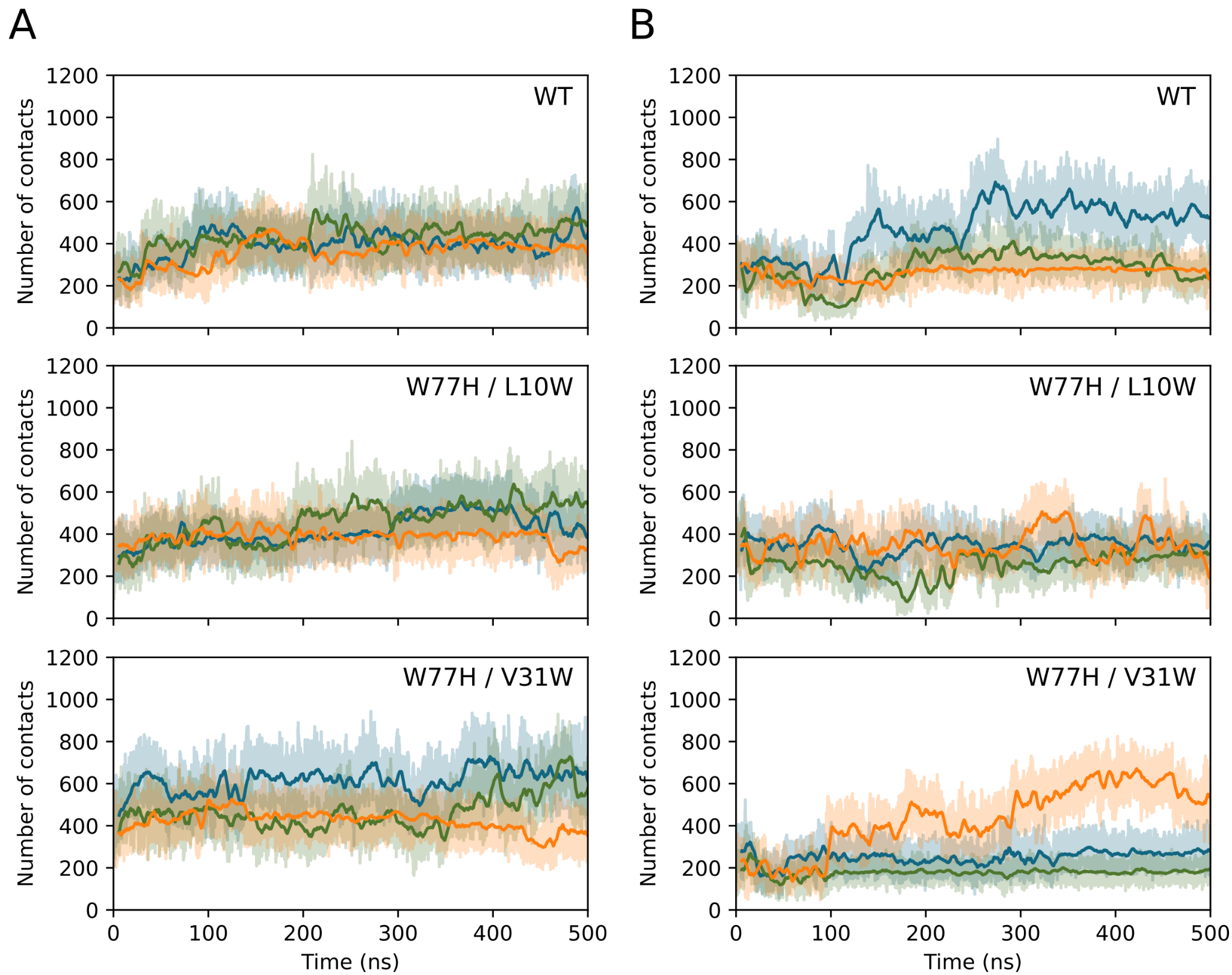
**

**Figure S5.** Time-series for number of atomic contacts (distance cut-off, r < 4.5 Å) for simulations of the Compact (**A**) and Extended (**B**) homodimer models of the wild-type (WT), together with the W77H/L10W and W77H/V31W mutants (as indicated the figure panels). The results from the three replicate simulations for each protein are presented in shades of blue, orange and green.


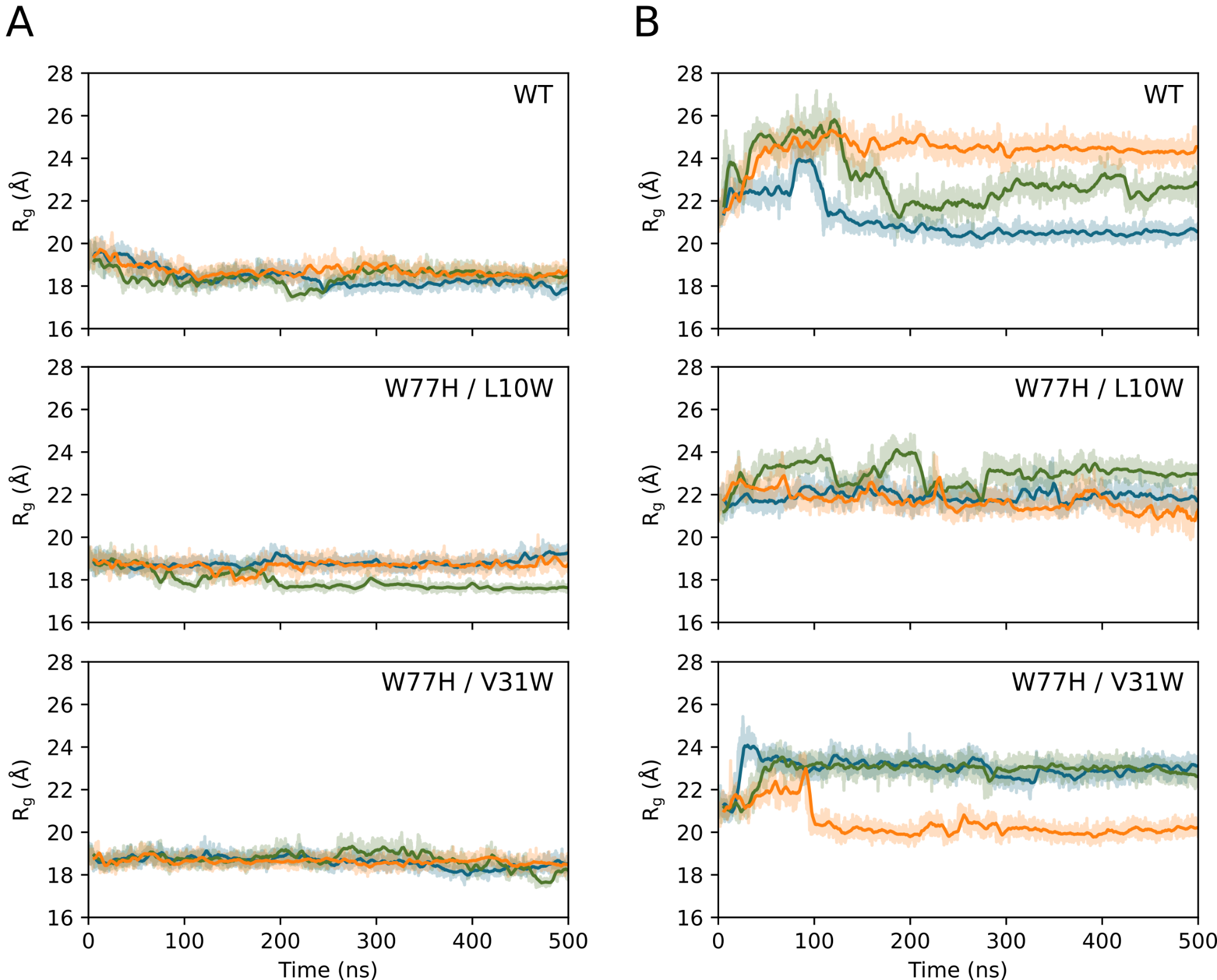


**Figure S6.** Time-series for Radius of gyration (R_g_) for simulations of the Compact (**A**) and Extended (**B**) homodimer models of the wild-type (WT), together with the W77H/L10W and W77H/V31W mutants (as indicated the figure panels). The results from the three replicate simulations for each protein are presented in shades of blue, orange and green.


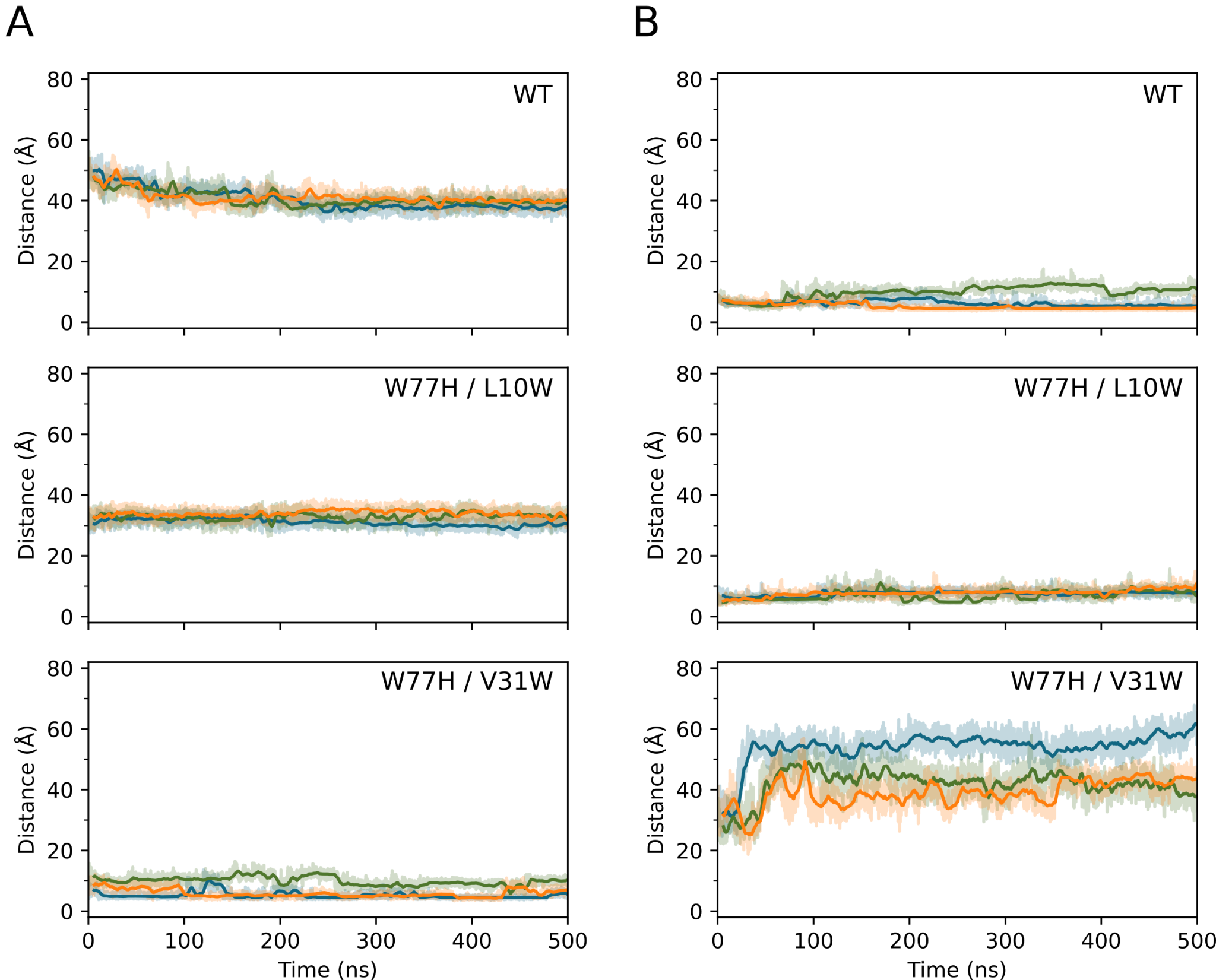


**Figure S7.** Time-series for the pair distance (D) between center of geometry of indole side chains for simulations of the Compact (**A**) and Extended (**B**) homodimer models of the wild-type (WT), together with the W77H/L10W and W77H/V31W mutants (as indicated the figure panels). The results from the three replicate simulations for each protein are presented in shades of blue, orange and green.


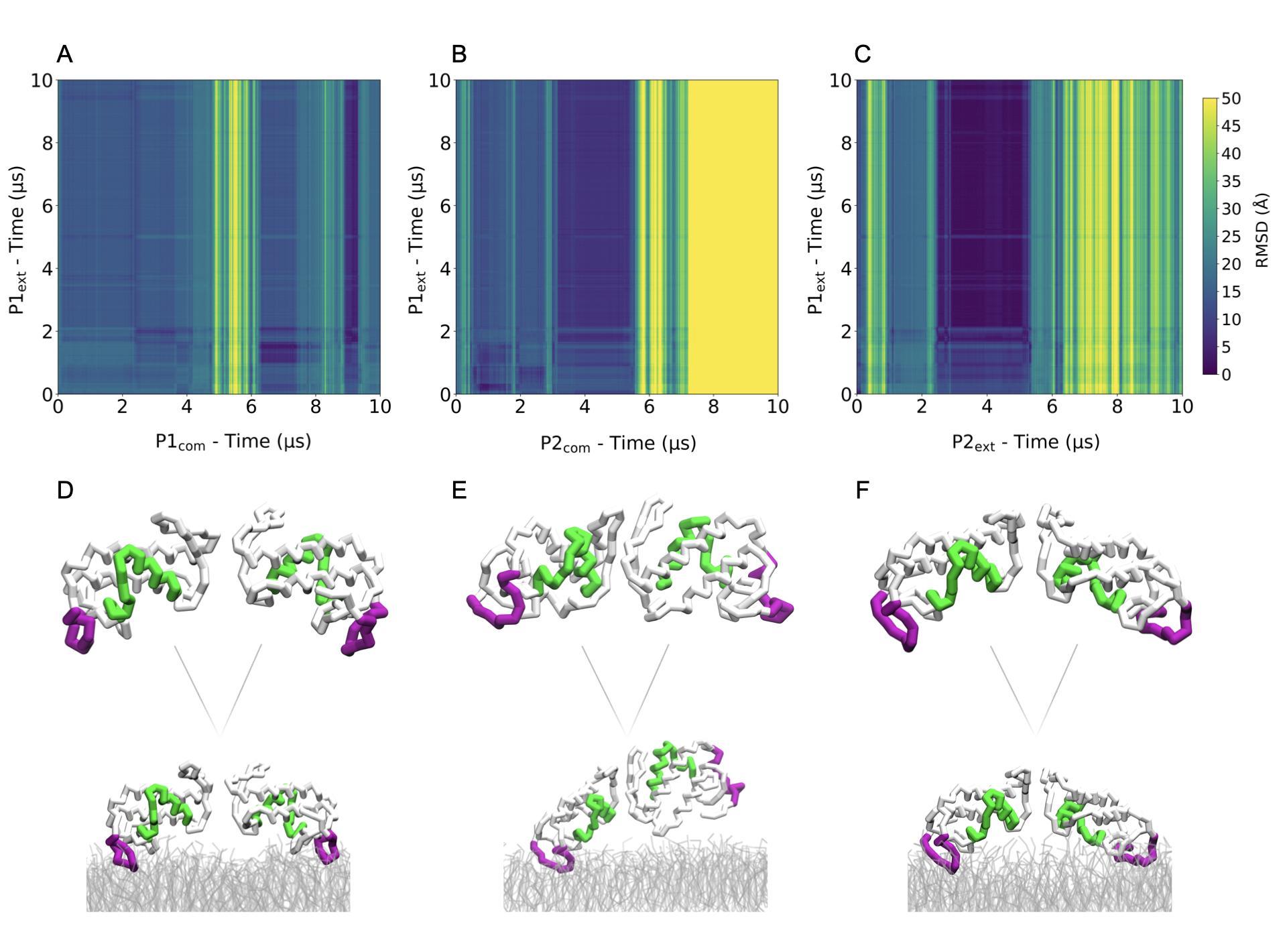


**Figure S8.** Comparison of the conformational ensembles sampled through CGMD simulations of the four different systems. Two-dimensional heatmaps of the root-mean-square deviation (RMSD) between protein backbone beads in the P1_ext_ trajectory and **(A)** P1_com_,  **(B)** P2_com_, and **(C)** P2_ext_ trajectories, respectively. The RMSD color scale ranges from 0 to 50 Å with darker purple corresponding to the lower RMSD values observed with higher structural similarity between the pair of dimers at the same times from the reference and the target trajectories. **(D–F)** Representative conformational pairs with low RMSD values, showing the recurrent membrane bound dimer configuration observed in all four simulations. These representative conformations correspond to time intervals at around 9 µs in the P1_com_ simulation **(D)**, 5 µs in the P2_com_ simulation **(E)**, and 4 µs in the P2_ext_ simulation **(F)**. The upper row of conformations are after structural superposition with the reference dimer to facilitate the structural comparison, whereas the lower row of structures depict their respective orientations relative to the membrane phospholipids (shown in gray). For clarity only the protein backbone is shown. In panels **D** and **F**, the C-terminal loop (in fuchsia) and hydrophobic IRS regions (in green) of both monomers are oriented toward the membrane, while in panel **E** these regions of only one monomer are associated with the membrane.

**
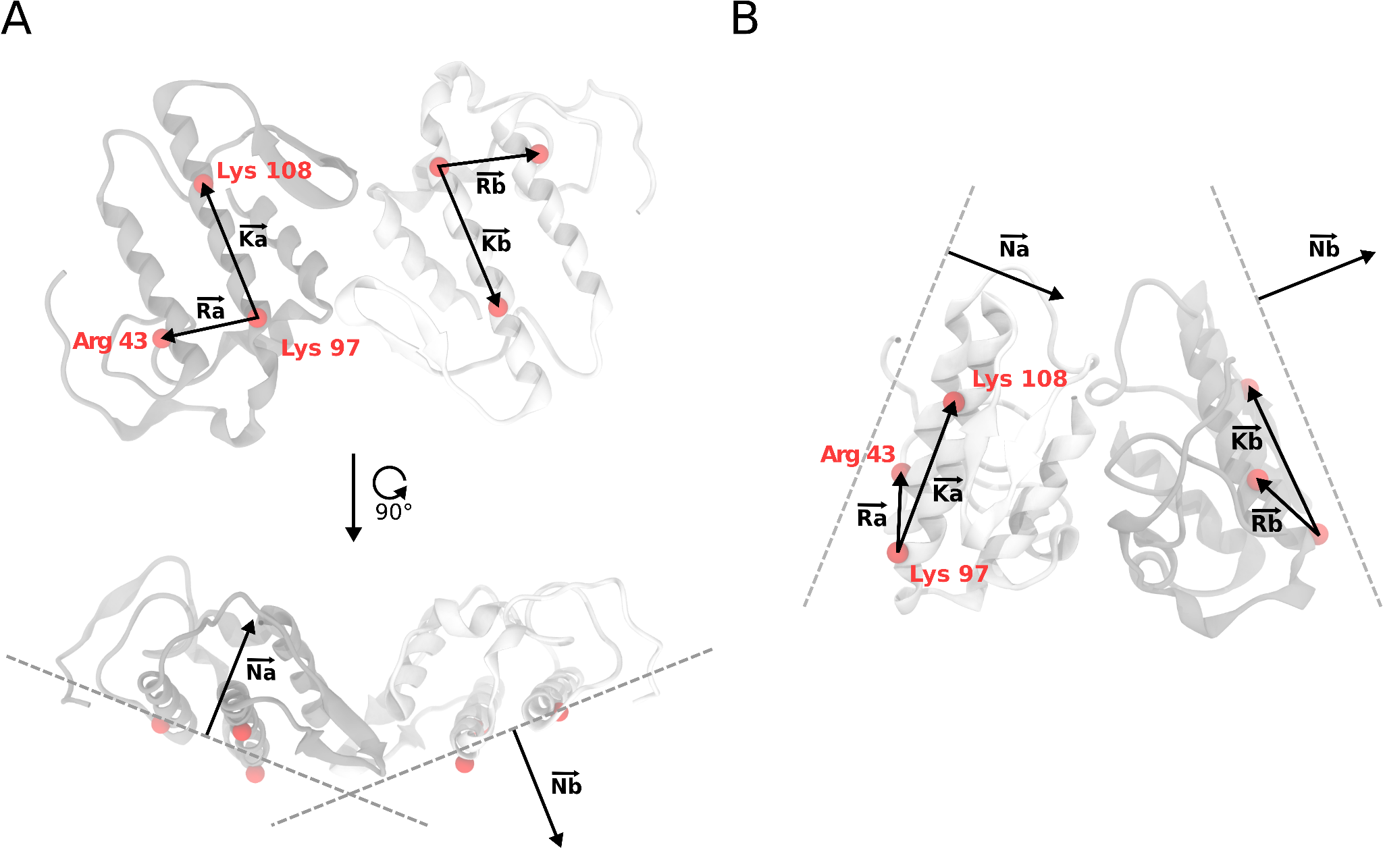
**

**Figure S9.** Representation of the vectors used to calculate azimuth (θ_A_) and tile (θ_T_) angles in the structures of the compact (**A**) and extended (**B**) BthTx-I homodimer conformations. The angle θ_A_ is defined by the vectors Ka and Kb, whereas the angle θ_T_ is defined between the vectors Na (calculated as Ra × Ka) and Nb (calculated as Kb × Rb). The angle θ_T_ represents the inter-monomer tilt, with higher values suggesting a more open conformation and lower values indicating a more closed state. The angle θ_A_ denotes the rotational orientation between the major α-helices of each monomer.


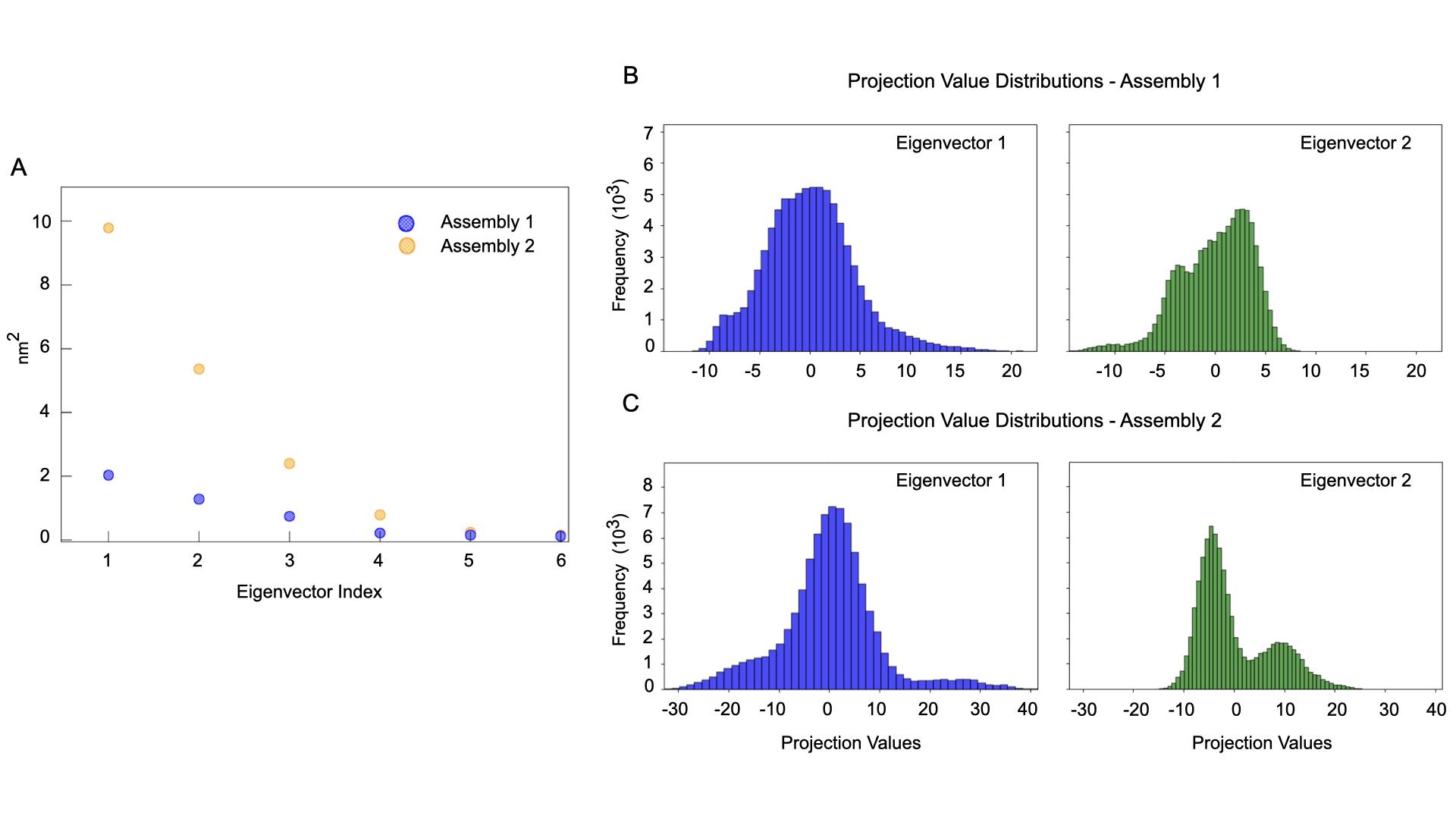


**Figure S10.** (A) Eigenvalues and the two first eigenvectors representative of most large amplitude motions sampled through the AT simulations of (B) Assembly 1 and (C) Assembly 2. Assembly 2 displays higher eigenvalues for the first few principal components, indicative of larger amplitude collective motions compared to Assembly 1. The latter shows a faster decay of eigenvalues, reflecting more restricted large scale dynamics consistent with the rigid conformational ensemble observed in the AT MD simulations. The collective motions associated with the two largest eigenvectors for Assembly 1 and Assembly 2 can be found as
